# Stable knockdown of Drp1 improves retinoic acid-BDNF-induced neuronal differentiation through global transcriptomic changes and results in reduced phosphorylation of ERK1/2 independently of DUSP1 and 6

**DOI:** 10.3389/fcell.2024.1342741

**Published:** 2024-03-14

**Authors:** Marvi Ghani, Peleg Zohar, Gyula Ujlaki, Melinda Tóth, Hailemariam Amsalu, Szilárd Póliska, Krisztina Tar

**Affiliations:** ^1^ Department of Medical Chemistry, Faculty of Medicine, University of Debrecen, Debrecen, Hungary; ^2^ Doctoral School of Molecular Medicine, University of Debrecen, Debrecen, Hungary; ^3^ Department of Biochemistry and Molecular Biology, Faculty of Medicine, University of Debrecen, Debrecen, Hungary

**Keywords:** DRP1, RNA seq, mitochondrial network rearrangement, *in vitro* neuronal differentiation, high-content analysis, ERK1/2, DUSP1 and DUSP6, huntingtin aggregates

## Abstract

**Background:** Dynamin-related protein Drp1 —a major mitochondrial fission protein— is widely distributed in the central nervous system and plays a crucial role in regulating mitochondrial dynamics, specifically mitochondrial fission and the organelle's shaping. Upregulated Drp1 function may contribute to the pathological progression of neurodegenerative diseases by dysregulating mitochondrial fission/ fusion. The study aims to investigate the effects of Drp1 on retinoic acid-BDNF-induced (RA-BDNF) neuronal differentiation and mitochondrial network reorganization in SH-SY5Y neuroblastoma cells.

**Methods:** We generated an SH-SY5Y cell line with stably depleted Drp1 (shDrp1). We applied RNA sequencing and analysis to study changes in gene expression upon stable Drp1 knockdown. We visualized the mitochondria by transmission electron microscopy and used high-content confocal imaging to characterize and analyze cell morphology changes and mitochondrial network reorganization during neuronal differentiation.

**Results:** shDrp1 cells exhibited fused mitochondrial ultrastructure with perinuclear clustering. Stable knockdown of Drp1 resulted in the upregulation of genes involved in nervous system development. High content analysis showed improved neurite outgrowth, segmentation, and extremities in differentiated shDrp1 cells. Neuronal differentiation was associated with a significant reduction in ERK1/2 phosphorylation, and ERK1/2 phosphorylation was independent of the dual specificity phosphatases DUSP1/6 in shDrp1 cells. Differentiated control underwent mitochondrial morphology remodeling, whereas differentiated shDrp1 cells retained the highly fused mitochondria and developed long, elongated structures. The shDrp1 cells responded to specific apoptotic stimuli like control *in vitro*, suggesting that Drp1 is not a prerequisite for apoptosis in SH-SY5Y cells. Moreover, Drp1 downregulation reduced the formation of toxic mHtt aggregates *in vitro*.

**Discussion:** Our results indicate that Drp1 silencing enhances RA-BDNF-induced neuronal differentiation by promoting transcriptional and mitochondrial network changes in undifferentiated cells. We also demonstrate that the suppression of Drp1 reduces toxic mHtt aggregate formation *in vitro*, suggesting protection against neurotoxicity. Thus, Drp1 may be an attractive target for further investigation in future strategies to combat neurodegenerative diseases.

## Introduction

Mitochondria are dynamic organelles that continuously fuse and divide, and the machinery that governs mitochondrial fission and fusion is conserved from yeast to humans (Okamoto and Shaw, 2005). The dynamic nature of the mitochondrial network is essential for aligning the capacity of the organelle with the energetic and metabolic requirements of eukaryotic cells (Okamoto and Shaw, 2005). Mitochondrial network adaptations are particularly evident in sperm flagellum, where fused mitochondria are wrapped around the base of the flagellum (Sutovsky et al., 1996), and during mitosis, when the mitochondrial network is highly fragmented (Taguchi et al., 2007). Regulated mitochondrial fission and fusion also play a role in organelle transport (e.g., in neurons, mitochondria quality control, and mitochondrial inheritance during cell division). Mitochondrial fragmentation is involved in programmed cell death programs (Otera et al., 2013) and participates in organelle quality control mechanisms by isolating defective, depolarized mitochondria and facilitates the removal of faulty organelles via mitophagy (Twig and Shirihai, 2011; Mao et al., 2013). It is also associated with mitochondrial dysfunction and disease development (Youle and van der Bliek, 2012; Guo et al., 2013). Unopposed fusion leads to hyperfused mitochondrial network with the role of protecting cells from metabolic insults (Mishra and Chan, 2016; Wai and Langer, 2016). Thus, proteins involved in regulating mitochondrial fission and fusion are crucial factors for maintaining the health of eukaryotic cells.

Mitochondrial fusion proteins include mitofusins (MFN1 and MFN2) and optic atrophy 1 (OPA1), and critical fission proteins include dynamin-related protein 1 (Drp1) and the mitochondrial fission protein 1 (Fis1) (Scott and Youle, 2010; Al Ojaimi et al., 2022). Mitochondrial fission is predominately regulated by Drp1 (Nunnari and Suomalainen, 2012). Drp1 is a large dynamin-related GTPase (Bleazard et al., 1999; Sesaki and Jensen, 1999) that cooperates with adaptor proteins to promote mitochondrial fission (Tieu et al., 2002). Drp1 is primarily cytosolic. However, Drp1 translocates to the mitochondrial surface to mediate the fission process. Due to its initial cytosolic location, mitochondrial receptors recruit and anchor Drp1 to the surface of the mitochondrial membrane. Fis1 is a mitochondrial receptor for Drp1 (Tieu et al., 2002; Suen et al., 2008). Other membrane receptors have been identified, including the mitochondrial fission factor Mff and the 49 and 51 kDa mitochondrial dynamics proteins Mid49 and Mid51, respectively (Koirala et al., 2013; Samangouei et al., 2018).

SH-SY5Y cells are neuroblast-like cells and are triple successive subclones of the parent SK-N-SH cell line, which was obtained from a bone marrow biopsy of a 4-year-old female neuroblastoma patient containing both neuroblast-like and epithelial-like cells (Ross et al., 1983). The cell line is a standard model in neuroscience. The SH-SY5Y cell line produces both substrate adherent (S-type) cells in a small portion and neuroblastic (N-type) cells that can undergo transdifferentiation (Ross et al., 1983). SH-SY5Y cells are characterized by their immortality, a stable karyotype of 47 chromosomes, and the ability to differentiate into mature human neurons (Forster et al., 2016; Shipley et al., 2016). Undifferentiated and differentiated SH-SY5Y cells differ in several ways (Riegerova et al., 2021). Undifferentiated cells rapidly proliferate and display short neuronal processes. In addition, undifferentiated cells are non-polarized, tend to form cell clumps and express neuronal markers indicative of immature neuronal cells. Differentiated SH-SY5Y cells exhibit extended, branched neuronal processes, may become polarized, and undergo less proliferation (Shipley et al., 2016).

*In vitro*, neuronal differentiation of SH-SY5Y cells can be induced by several methods, including treatment with all-trans retinoic acid (RA) (Truckenmiller et al., 2001; Korecka et al., 2013). The signaling pathways involved in neuronal differentiation of SH-SY5Y cells after RA treatment have been extensively studied (Lopez-Carballo et al., 2002; Lee and Kim, 2004; Miloso et al., 2004; Attoff et al., 2020). PI3K/Akt, JNK, and p35/CDK-5 play essential roles in neurite outgrowth, cell cycle arrest, and differentiation in response to RA treatment (Lopez-Carballo et al., 2002; Yu et al., 2003; He et al., 2009). Furthermore, Akt is involved in RA-mediated neuroprotection from 6-OHDA in SH-SY5Y cells (Cheung et al., 2009). Miloso et al. reported that RA-induced neuritogenesis of SH-SY5Y cells is PKC-dependent and ERK-independent (Miloso et al., 2004). In contrast, Singh et al. demonstrated the involvement of transglutaminase in RA-induced differentiation of SH-SY5Y cells. In the Singh study, activating Rho by transamidation promoted the activation of ERK1/2 and the p38γ MAP kinase. These observations suggest that RA activates different signaling pathways with diverse roles during neuronal differentiation (Singh et al., 2003). The effects of RA can be enhanced with the brain-derived neurotrophic factor (BDNF). BDNF is a member of the neurotrophin family of growth factors. RA differentiation induces the expression of tropomyosin-related kinase B (TrkB) receptor, which turns cells responsive to BDNF. BDNF regulates synapses, maturation, and plasticity of dendrites in young neurons through TrkB signaling, stimulating tyrosine phosphorylation of TrkB (Kaplan et al., 1993; de Medeiros et al., 2019). The BDNF-TrkB interaction activates three signaling pathways: the phospholipase-Cγ (PLCγ) pathway, the phosphatidylinositol 3-kinase (PI3K) pathway, and the ERK-MAPK family. All three pathways modulate synaptic plasticity, neuronal differentiation, and neuroprotection through regulating CREB, a transcription factor. Furthermore, PI3K regulates dendritic growth via Akt and mTOR (Schiro et al., 2022). Recent studies have demonstrated that BDNF triggers the expression of mature neuronal markers and molecular polarization and results in the differentiation of mature neurons from SH-SY5Y cells with distinct neuronal morphology (Hromadkova et al., 2020; Riegerova et al., 2021).

RA-BDNF-induced neuronal differentiation of SH-SY5Y cells was optimized by Forster et al., 2016. Differentiated cells show reduced expression of oxidative stress response genes, increased intracellular ATP levels, and reduced mitochondrial membrane potential. During neuronal differentiation, cells transition from an oxidative stress-resistant state to a neural cell with increased energy stress and oxidative vulnerability. Thus, differentiated SHSY5Y cells are more suitable for studying neuronal energetic vulnerability than undifferentiated SH-SY5Y cells. Another advantage of the RA-BDNF optimized differentiation protocol is the increased selective pressure against S-type cells and a reduced duration of differentiation. Thus, a homogenous cell population can be obtained to perform high-content analysis studies.

Balanced mitochondrial dynamics are essential for neuronal differentiation, development, and function (Vantaggiato et al., 2019). During neurogenesis, mitochondria undergo morphological changes from a mixture of tubular structures to a more elongated and hyperfused meshwork, illustrating maturation and allowing metabolic adaptation from glycolysis to mitochondrial respiration for higher energy demand (Beckervordersandforth et al., 2017; D'Aloia et al., 2024; Khacho and Slack, 2018). Metabolic reprogramming was confirmed during neuronal differentiation in primary cortical neurons and neural progenitor cells (Agostini et al., 2016; Zheng et al., 2016).

Drp1 plays a central role in the nervous system, and upregulated Drp1 function may contribute to the pathological progression of neurodegenerative diseases by dysregulating mitochondrial fission/fusion (Wang et al., 2009; Su et al., 2010; Guo et al., 2013). Imbalanced mitochondrial dynamics is a crucial event underlying the pathology of Huntington’s disease-induced neurotoxicity. In Huntington’s disease, a mutation in the huntingtin gene results in the expression of mutant huntingtin protein (mHtt), which has abnormal polyQ expansions. The mutant protein is localized to the mitochondrial membranes, which induces free radical production, reduces ATP production, and causes cell death (Beal, 2005; Reddy, 2008). In primary cultured neurons of transgenic HD mice and human post-mortem brains, mHtt induces NO overproduction, leading to S-nitrosylation of Drp1. S-nitrosylated Drp1 triggers mitochondrial fragmentation, synaptic damage, and neuronal loss (Haun et al., 2013). In addition, mHtt disrupts calcium homeostasis, increases intracellular calcium levels, and stimulates calcium-dependent phosphatase calcineurin, which dephosphorylates Drp1, rendering it active (Costa et al., 2010).

This study aims to determine the role of Drp1 in RA-BDNF-induced neuronal differentiation of SH-SY5Y cells and characterize differentiated neurons using high-content analysis. Our transcriptomic analysis demonstrates that stable knockdown of Drp1 results in the upregulation of genes involved in nervous system development, such as synapse assembly, neurogenesis, differentiation, and morphogenesis, suggesting an involvement in the nuclear transcriptional program. Our results show that neuronal differentiation, including neurite outgrowth, segmentation, and the number of extremities, is enhanced in cells stably depleted of Drp1 (shDrp1) compared to control. RA-BDNF-induced neuronal differentiation is associated with a significant reduction of ERK1/2 phosphorylation, suggesting ERK1/2 independent RA-induced differentiation in shDrp1 cells. Furthermore, the phosphorylation level of ERK1/2 is independent of the dual specificity phosphatases DUSP1/6 in shDrp1 cells. Control cells undergo mitochondrial morphology remodeling, while shDrp1 cells conserve highly fused mitochondria in addition to the development of long, elongated tubules. The shDrp1 cells respond to specific apoptotic stimuli like control *in vitro*, suggesting that Drp1 is not a prerequisite for apoptosis in SH-SY5Y cells.

We also show that the toxic aggregate formation of mHtt following the overexpression of the N-terminal mHtt fragments is reduced. Our results indicate that lowering Drp1 promotes transcriptional changes and improves neuronal differentiation. RA-BDNF-induced differentiation of Drp1 knockdown cells was associated with diminished ERK1/2 activation. Moreover, we show that the suppression of Drp1 reduces toxic mHtt aggregate formation *in vitro,* suggesting protection against neurotoxicity.

## Materials and methods

All materials were purchased from Sigma-Aldrich unless otherwise specified.

### Cell culture

Modified human SH-SY5Y (European Tissue Culture) cells were maintained in DMEM with high glucose, supplemented with 10% fetal bovine serum (FBS), 2 mM L-glutamine, and 1x (vol/vol) antibiotic-antimycotic (Gibco), at 37°C in a 5% CO_2_ incubator. HEK293T cells were cultured in DMEM supplemented with 10% FBS, 2 mM L-glutamine, and 1x (vol/vol) antibiotic-antimycotic (Gibco) at 37°C in a 5% CO_2_ incubator.

### Downregulation of *hDNM1L*/hDrp1

We used lentiviral technology to downregulate the expression of h*DNM1L/*hDrp1, according to Douida et al., 2020. One day before transfection, HEK293T cells were seeded to reach 80% confluency the next day in a 24-well plate. The expression plasmid pGIPZ-GFP containing the target sequences and the control plasmids were obtained from the shRNA Facility of the Albert Einstein College of Medicine, Bronx, NY, United States, and were provided by Dr. Marion Schmidt. All information on the plasmids is listed in Supplementary Table S1. The packaging and enveloping vectors (HDM-Hgpm2, RC-CMV/Rev, HDM-tat1b, and HDM-VSV-G) were courtesy of Dr. Orsi Giricz (Albert Einstein College of Medicine, Bronx, NY, United States). Transfection was performed using Lipofectamine 3000 (ThermoFisher Sci.) according to the manufacturer’s protocol. Four replicates (wells) were used for each expression plasmid containing the target sequences. The HDM-MIX consisted of HDM-Hgpm2, RC-CMV/Rev, HDM-tat1b (25 ng/μL each), and 50 ng/μL of HDM-VSV-G. The HDM-MIX and the expression plasmids (100 ng/μL) were co-transfected at 1:1. After 24 and 48 h, the transfected cells were visualized by fluorescent microscopy to confirm GFP expression. Culture media was collected 48 and 96 h after transfection. Virus-containing media was filtered through 0.45-µm pore filters and immediately used for transduction. For human SH-SY5Y neuroblastoma cell transduction, cells were incubated with the virus containing antibiotic-free media supplemented with 8 μg/mL polybrene. GFP expression in cells was monitored every day under a fluorescent microscope. Selection with 1.25 μg/mL puromycin was started 72 h after viral transduction. The puromycin-selected and amplified cells were further analyzed by real-time PCR and western blot to verify *hDNM1L*/hDrp1 depletion.

### Titration of antibiotic selection

Puromycin was used to determine the optimal concentration for selecting transduced target cells to generate stable transduced target cell lines. SH-SY5Y cells were seeded to reach 90% confluency in 6-well plates. The appropriate complete medium was added, containing 0, 0.5, 1, 1.25, 2.5, or 5 μg/mL puromycin (Gibco). Cells were incubated for 10 days, replacing the selection media every other day. Cells were monitored using a light microscope every day. The lowest puromycin concentration (1.25 μg/mL) that induced massive cell death in 7 days and killed all cells within 10 days was used for further selection and to maintain the stable cell line under antibiotic selection pressure.

### Neuronal differentiation

The differentiation of SH-SY5Y cells was performed according to Forster et al. (Forster et al., 2016) with a slight modification. DMEM (ThermoFisher Sci.) with high glucose (25 mM), 4 mM L-glutamine, and 1% penicillin/streptomycin (P/S), without sodium pyruvate, was used for phase 1 (days 0–3) culture medium. The medium was supplemented with 10 µM RA and 3% heat-inactivated FBS. Neurobasal-A medium without phenol red (ThermoFisher Sci.), supplemented with 2 mM L-glutamine, 1x (v/v) N-2 supplement 100× (ThermoFisher Sci.), 1% P/S, and human BDNF (Peprotech) at a final concentration of 50 ng/mL was used for phase 2 (days 3–6) differentiation. Cells were collected or analyzed at Day 0 (undifferentiated) and after completing phase 1 (Day 3) and phase 2 (Day 6) differentiation.

### Tubulin tracker deep red staining for live cell high content analysis confocal imaging

To stain neuron-specific β-tubulin III, 96-well microplates (Corning, Merck) were coated with 5 μg/cm^2^ laminin at 37°C for 2 hours before seeding 5000 cells per well. The control and shDrp1 neuroblastoma cells were differentiated using the neuronal differentiation protocol (Section 2.4). On Day 6, cells were stained with 1 μΜ Tubulin Tracker Deep Red (ThermoFisher Sci.) to detect neurite outgrowth and 10 μM Hoechst 333642 for nuclei staining for 30 min at 37°C in 5% CO_2_. Cells were washed twice with Hanks’ Balanced Salt Solution containing calcium and magnesium (ThermoFisher Sci.). Images of live cells were acquired at 37°C and 5% CO_2_ using an Opera Phenix High Content Analysis system (PerkinElmer).

### Neurite analysis

Automated confocal microscopy was performed on an Opera Phenix High Content Analysis system (PerkinElmer). Image-acquisition settings were ×40 water objective (NA = 1.1), appropriate lasers, and filters for Hoechst, eGFP, and Tubulin Tracker Deep Red in sequential mode to exclude spectra overlap. Neurites were detected with a 16-bit camera under nonsaturating conditions. Quantitative image analysis was performed with the built-in software (Harmony 4.9, PerkinElmer). Cell segmentation was performed based on Hoechst and eGFP staining to detect the nuclei and cytoplasm. Analysis was performed with the “CSIRO neurite Analysis 2” built-in method of the Harmony 4.9 software (PerkinElmer) (Wang et al., 2010). The true nuclei were defined based on the Hoechst channel. Neurites were detected using the Tubulin Tracker Deep Red signal with the “Find-Neurites” building block. The length, width, and area units were µm, µm, and µm^2^, respectively. Other parameters were defined by the HCS built-in functions of the software and presented as arbitrary units. The values are presented as mean ± SD and normalized to cell numbers based on true nuclei. The parameters for neurite outgrowth analysis, which are summarized in Supplementary Table S2, were chosen according to the “CSIRO neurite Analysis 2” built-in method and Wang et al., 2010.

### RNA extraction

Total RNA was extracted using TRI reagent (Molecular Research Center, Inc.) following the manufacturer’s protocol. Samples were treated with DNase I in DNA digestion buffer for 15 min before reverse transcription (Zymo Research). For cDNA synthesis, a High-Capacity cDNA Reverse Transcription Kit (Applied Biosystems) was used to reverse transcribe 1 μg of total RNA with random primers. The quality of the cDNA was checked on a 1% agarose gel.

### Quantitative real-time PCR

Real-time PCR was performed with a LightCycler 480 Thermocycler (Roche) using Xceed qPCR Probe 2 × Mix Hi-ROX (Institute of Applied Biotechnologies, IAB) according to the manufacturer’s protocol. Cycling conditions were as follows: initial denaturation 95°C for 30 s, 1 cycle; PCR 95°C for 5 s and 60°C for 30 s, 40 cycles; and melt curve analysis 95°C for 0 s, 65°C for 15 s and 95°C for 0 s, cooling 50°C for 30 s, 1 cycle. Threshold values (*C*_t_ values) for all biological replicates (n ≥ 3) were normalized to GAPDH and PPIA**.** Each biological replicate included at least three technical replicates for each gene. The fold change in gene expression compared to respective undifferentiated and differentiated cells was calculated using the 2^−ΔΔCT^ method (Livak and Schmittgen, 2001). The primers are shown in Supplementary Table S3.

### Western blotting

Cells were washed with 1 × phosphate-buffered saline (PBS) and lysed in RIPA buffer (50 mM Tris-HCl pH 7.4, 150 mM NaCl, 0.5% Na-deoxycholate, 2 mM EDTA, 1% NP-40, and 50 mM NaF) supplemented with a protease inhibitor cocktail (1 mM benzamidine, 1 mM PMSF, and cOmplete Mini-EDTA-free protease inhibitor cocktail (Merck)). Cells were centrifuged at 12,000 rpm at 4°C for 15 min. The supernatants were collected, and the protein concentration was estimated using a Bradford protein assay. Proteins (20 μg/well) were resolved on standard SDS-polyacrylamide gel electrophoresis (PAGE) and transferred to a nitrocellulose membrane (GE Healthcare Life Sciences). Membranes were incubated with primary antibodies overnight at 4°C and subsequently incubated with secondary antibodies for 2 h at room temperature (RT). Blots were developed with enhanced chemiluminescence (Santa Cruz Biotechnology). The list of antibodies and dilutions is provided in Supplementary Table S4.

### Mitochondrial staining for live cell high content analysis confocal imaging

To stain the mitochondria, 96-well microplates (Corning, Merck) were coated with 5 μg/cm^2^ laminin at 37°C for 2 hours before seeding 5000 cells per well. The control and shDrp1 neuroblastoma cells were differentiated using the neuronal differentiation protocol (Section 2.4). On Day 6, cells were carefully rinsed with 1 × PBS and stained with 100 nM Mitotracker Red CMX ROS (ThermoFisher Sci.) and 10 µM Hoechst 33342 in serum-free media for 20 min at 37°C in a 5% CO_2_ incubator. The media was changed to fresh culture media, and live cell imaging was performed using an Opera Phenix High-Content-Screening system (PerkinElmer) at 37°C at 5% CO_2_. Image-acquisition settings were ×63 water objective (NA = 1.15), appropriate lasers, and filters for Hoechst, eGFP, and Mitotracker Red in sequential mode to exclude spectra overlap. Detection was performed with a 16-bit camera under nonsaturating conditions. Cell segmentation was performed based on Hoechst and EGFP staining to detect the nuclei and cytoplasm. Mitochondria were determined by the Mitotracker Orange signal using the Find-Spots building block. Mitochondria morphology analysis was performed as previously described (D'Aloia et al., 2024; Douida et al., 2021).

### Seahorse XF analysis

Oxygen consumption rate (OCR) was measured at Days 0 and 6 of control and shDrp1 cells. Cells were seeded in appropriate culture media in XF96 cell culture microplates (Agilent, Seahorse Bioscience) with proper background correction wells and incubated overnight at 37°C in a 5% CO_2_ incubator. On the following day (Day 0), the media were changed to phase 1 media in cells subjected to phase 1 (days 0–3) and 2 differentiations. On Day 3, the media was replaced with phase 2 media in cells subjected to phase 2 (days 3–6) differentiation. The sensor cartridge was prepared by adding 200 µL of Seahorse Bioscience XF96 calibrant solution (pH, 7.4) (Agilent, Seahorse Bioscience) to each Seahorse Bioscience 96-well utility plate well. The sensors with the calibrant solution were incubated overnight at 37°C without CO_2_. Measurements were performed using the Seahorse XF96 Analyzer. For XF Cell Mito Stress analysis, the media was replaced with 180 µL of XF assay medium (Agilent, Seahorse Bioscience) supplemented with 2 mΜ L-glutamine and 25 mM glucose on the day of measurement, and the plate was incubated at 37°C without CO_2_ for 1 h. After a 20-min equilibration time, OCR was measured every 6 min (1 min mixing, 5 min measurement) for five cycles. Mitochondrial inhibitors were applied at the following final concentrations: 1.5 µM oligomycin (O), 1 µM FCCP (F), 1 µM antimycin-A (A), and 1 µM rotenone (R). The OCR values were normalized to the total protein concentration of each well using a quick Bradford protein assay (BIO-RAD). Data were analyzed using a 2.3 Agilent Seahorse Desktop Software. Reports were generated by the Multi-File XF Report Generator (Agilent, Seahorse Bioscience) that automatically calculates assay parameters of the Seahorse XF Cell Mito Stress Test as the average ± S.E.M of the average parameter value of the biological replicate groups.

### Sulphorhodamine B assay

Cell viability after RA treatment was measured using a sulphorhodamine B (SRB) assay. The assay is based on measuring cellular protein content, as described by Vichai & Kirtikara (Vichai and Kirtikara, 2006). Cell viability was calculated as follows: % cell viability = Absorbance sample/Absorbance negative control or untreated sample × 100.

### Propidium iodide staining to assess cell viability

Cell viability of undifferentiated and differentiated control and shDrp1 cells on Days 3 and 6 was assessed using 500 ng/mL propidium iodide (PI) (BD Biosciences) counterstained with 10 μM Hoechst 33342. Briefly, the media was aspirated, and culture media containing 500 ng/mL PI and 10 μM Hoechst was added, and cells were incubated for 15 min at 37°C in a 5% CO_2_ incubator. Live cell imaging was performed using an Opera Phenix High-Content-Screening confocal microscopy (PerkinElmer) at 37°C with 5% CO_2_. Image-acquisition settings were ×10 objective (NA = 0,3), appropriate lasers, and filters for Hoechst and Alexa 568 for PI in sequential mode to exclude spectra overlap. A 16-bit camera under nonsaturating conditions was used for detection. Analysis was performed using the “Live/Dead Cells” built-in method of the Harmony 4.9 software (PerkinElmer). The true nuclei were defined based on the Hoechst channel. The PI-positive cells were selected using the channel Alexa 568. The formula output to calculate the percentage of dead cells was as follows: cells stained positive for Alexa Fluor 568/true nuclei ×100.

### Immunofluorescence staining

Control and shDrp1 cells (3.5 × 10^4^ cells/well) were seeded in a 12-well plate. The cells were differentiated on the following day (Section 2.4). Undifferentiated and differentiated cells on Days 3 and 6 were gently rinsed twice with 1 × PBS, fixed with 4% paraformaldehyde (PFA) for 15 min, rinsed with 1× PBS, and permeabilized with 0.01% Triton X-100 for 15 min. The cells were blocked with 5% BSA in 1 × PBS for 1 hour and incubated with the primary anti-Tuj1 antibody (Biolegend) at 1:1000 dilution in 1 × PBS for 90 min at RT. The samples were gently rinsed with 1 × PBS and incubated with the secondary antibody Alexa Fluor 568 (ThermoFisher Sci.) and Hoechst 33342 at 1:1000 dilution for 1 h at RT. Moviol 4-88: Dabco 33-LV (1:50) was used to mount the coverslips on the slides. Images were acquired on a Leica SP8 confocal laser scanning microscope.

7000 control or shDrp1cells/well were seeded on Perkin Elmer 96-well microplates coated with 5 μg/cm2 laminin for transient transfections. On the following day, cells were transiently transfected with pHM6-Q23 (a gift from David Rubinsztein, Addgene plasmid #40263; http://n2t.net/addgene:40263; RRID: Addgene_40263) (Narain et al., 1999) expressing the wild-type N-terminal huntingtin fragment (N-Htt) or pHM6-Q74 (a gift from David Rubinsztein, (Addgene plasmid # 40264; http://n2t.net/addgene:40264; RRID: Addgene_40264) (Narain et al., 1999), expressing the mutant N-Htt huntingtin fragment, using Lipofectamine 3000 according to the manufacturer’s protocol and Aladdin et al. (Aladdin et al., 2020). The cells were incubated for 72 h and gently rinsed twice with 1× PBS. The cells were fixed with 4% PFA for 15 min, rinsed with 1× PBS, and permeabilized with 0.01% Triton X-100 for 15 min. After blocking with 5% BSA in 1× PBS for 1 hour, the cells were incubated with the primary anti-HA-Tag antibody (1:1000 dilution, Cell Signaling) for 90 min at RT. The samples were gently rinsed with 1 × PBS and incubated with secondary antibody Alexa Fluor 568 (ThermoFisher Sci.) and Hoechst 33342 at 1:1000 dilution for 1 hour at RT. Images were acquired on an Opera Phenix High Content Screening System (PerkinElmer). Image-acquisition settings were ×40 water objective (NA = 1.1), appropriate lasers, and filters for Hoechst, eGFP, and Alexa 568 in sequential mode to exclude spectra overlap. Detection was performed with a 16-bit camera under nonsaturating conditions.

Images were analyzed using Harmony 4.9 software (PerkinElmer, Waltham, MA, United States). The nuclei were segmented on the Hoechst channel, and the object’s borders were extended on the eGFP channel. The insoluble huntingtin protein fragments were segmented on the Alexa568 channel using the software’s embedded spot analysis module. The values are expressed as means ± SDs.

### Transmission electron microscopy

Monolayers of control and shDrp1 cells were grown on Aclar thermoplastic film (EMS- Electron Microscopy Sciences). Cell pellets were fixed in 3% glutaraldehyde dissolved in 0.1 M cacodylate buffer (pH, 7.4), containing 5% sucrose for 1 h at RT. After washing the cells several times in cacodylate buffer (pH, 7.4), samples were post-fixed in 2% osmium tetroxide dissolved in 0.1 M cacodylate buffer (pH, 7.4) for 1 h at RT. Following several washes in cacodylate buffer (pH, 7.4), cells were dehydrated and embedded into Durcupan ACM resin. Ultrathin sections were collected on Formvar-coated single-slot grids and counterstained with uranyl acetate and lead citrate. Sections were observed with a transmission electron microscope (JEOL 1010) and photographed at a magnification of 4000–10000 × with an Olympus Veleta CCD camera.

Cell and mitochondria contours were manually segmented. Only mitochondria with visible and intact internal membranes were considered for consecutive analyses. Amira 3D (version 2022.1; ThermoFischer Scientific) image analysis software was used to analyze the numerical parameters of the segmented structures as follows: visible area (μm^2^) of the cell in a given image, the total (summed) and average area (μm^2^) and inside length (μm) of mitochondria within the visible part of the cell. The length (inside length; μm) and area (μm^2^) were determined for 13-76 individual mitochondria/cell in control and 12-122 individual mitochondria/cell in the shDrp1 cell line.

The clustering of mitochondria was quantified as a measure of the closest neighbor of each mitochondrion in μm. Mitochondria were color-coded based on their distance from the closest neighbor (Garza-Lopez et al., 2021). Distance values in μm were extracted using the Label Analysis module of Amira 3D. Then, these distance values were displayed using the Colorize by Measure module of Amira 3D, which colored the mitochondria according to the values. Color scales were added using the Colormap Legend module from Amira 3D.

### RNA-seq

High throughput mRNA sequencing analysis was performed on an Illumina sequencing platform to obtain global transcriptome data. Total RNA was extracted from control and shDrp1 cells grown in T-75 flasks up to 90% confluence (Zymo Research). The quality of total RNA was checked on an Agilent BioAnalyzer using a Eukaryotic Total RNA Nano Kit according to the manufacturer’s protocol. Samples with an RNA integrity number (RIN) value of >7 were accepted for the library preparation process. RNA-Seq libraries were prepared from total RNA using an Ultra II RNA Sample Prep kit (New England BioLabs) according to the manufacturer’s protocol. Briefly, poly-A RNAs were captured by oligo-dT conjugated magnetic beads. The mRNAs were eluted and fragmented at 94°C. First-strand cDNA was generated by random priming reverse transcription. After the second-strand synthesis step, double-stranded cDNA was generated. After repairing ends, A-tailing, and adapter ligation steps, adapter-ligated fragments were amplified in enrichment PCR. The libraries were sequenced on an Illumina NextSeq 500 instrument using single-end 75-cycle sequencing.

### RNA-seq data analysis

Raw sequencing data (fastq) was aligned to the human reference genome version GRCh38 using the HISAT2 algorithm, and BAM files were generated. Downstream analysis was performed using StrandNGS software (www.strand-ngs.com). BAM files were imported into the software DESeq algorithm used for normalization. CytoScape 3.4.0 with the ClueGo application was used for gene ontology analysis of differentially expressed genes.

### Statistical analysis

Data are presented as means and standard deviations (SDs) of n ≥ 3 experiments. The normality of the population was determined using the Shapiro-Wilk test. One-way ANOVA with multiple comparisons followed by Tukey *post hoc* tests or Kruskal-Wallis test followed by Dunn’s multiple comparison *post hoc* test, and unpaired *t*-test or Mann-Whitney test were used for analyses (Torocsik et al., 2021). Two-way ANOVA with Tukey *post hoc* test was used to analyze the Seahorse experiments and mitochondrial classes. Statistical analyses were conducted using GraphPad Prism v9.5.1. A *p*-value of <0.05 was considered significant.

For RNA-seq data analysis, differentially expressed genes were determined by a moderated *t*-test with Bejamini-Hochberg FDR for multiple testing corrections. A hypergeometric test with Bonferroni step-down correction was used to determine overrepresented GO categories. The Bonferroni step-down corrected *p*-value <0.05 was considered significant.

## Results

### Stable knockdown of Drp1 causes fused mitochondrial ultrastructure with perinuclear clustering and global transcriptomic changes in SH-SY5Y cells

**FIGURE 1:**
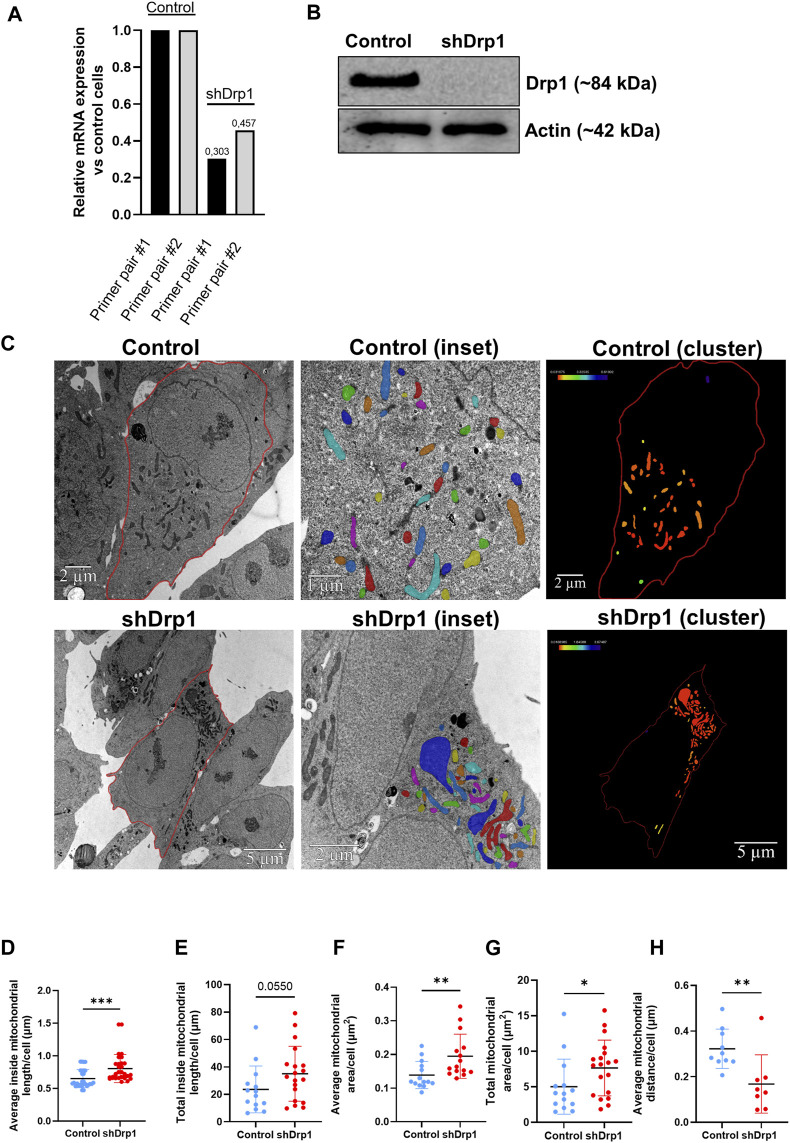
Generating a stably depleted human neuroblastoma cell line for Drp1 using lentiviral technology. Using lentiviral technology, DNM1L/Drp1 expression was downregulated in the SH-SY5Y human neuroblastoma cell line. HEK293T cells were transfected with the mixture of packaging-enveloping vectors and the plasmid pGIPZ-GFP containing the shRNA target sequence to produce the virus. Cells stably expressing empty pGIPZ-GFP were used as a control. Transduction of SH-SY5Y neuroblastoma cells was conducted using 8 μg/mL polybrene, and cells were maintained in media containing 1.25 μg/mL puromycin for selection. **(A)** The qPCR analysis shows successful silencing of *DNM1L* in shDrp1 cells. The sequences of primer #1 and #2 are listed in Supplementary Table S3. **(B)** Total cell lysates from control and shDrp1 cells were separated by SDS-PAGE to analyze Drp1 protein levels. Actin was used as the loading control. **(C)** Representative transmission electron microscopy images of mitochondria in control (upper panel) and shDrp1 cells (lower panel). Cell and mitochondria contours were manually segmented. Only mitochondria with visible and intact internal membranes were considered for consecutive analyses (insets). Mitochondria were displayed using a default shared colormaps option of Amira 3D with 8 distinct colors. Colors are independent of values; their purpose is to show the mitochondria used for further analyses. Amira 3D (version 2022.1; ThermoFischer Scientific) image analysis software was used to analyze the numerical parameters of the segmented structures. The clustering of mitochondria was quantified as a measure of the closest neighbor of each mitochondrion. Mitochondria were color-coded based on their distance from the closest neighbor. Distance values in μm were extracted using the Label Analysis module of Amira 3D. These distance values were displayed using the Colorize by Measure module of Amira 3D, which colored the mitochondria according to the values. Color scales were added using the Colormap Legend module from Amira 3D. A scale bar was added by the software to the upper right corner of each image. Quantitative analyses of average inside mitochondrial length (μm) **(D)**, total inside mitochondrial length (μm) **(E)**, average **(F)** and total **(G)** mitochondrial area (μm^2^), and average mitochondrial distance (μm) **(H)**. Mann-Whitney test was used for statistical analyses. Only *p* values *p* < 0.05 are considered statistically significant.

A human neuroblastoma SH-SY5Y cell line stably depleted of Drp1/DNML1 was generated as described in our previously published manuscript (Douida et al., 2020). qRT-PCR confirmed knockdown efficiency with two primer pairs and Western blotting (Figures 1A,B). The relative changes in the DNM1L (gene name for Drp1) gene expression using the two primer pairs confirmed efficient knockdown. As a control, SH-SY5Y cells were stably expressing pGIPZ-GFP (referred to as control). Cells stably depleted of Drp1 are referred to as shDrp1.

Stable depletion of Drp1 promotes changes in mitochondrial morphology, resulting in an elongated, hyperfused mitochondrial network (Kitamura et al., 2017). To confirm the effects of stable knockdown in our cellular model, we performed transmission electron microscopy (TEM) to examine the ultrastructure of mitochondria in both control and shDrp1 cells (Figure 1C). TEM images revealed that shDrp1 cells contained long, highly fused, clustered, and elongated mitochondrial networks compared to the control cells (Figure 1C). We measured inside mitochondrial length, area and the formation of mitochondrial clusters using the Amira 3D analysis software. The average inside mitochondrial length (Figure 1D) and area (Figure 1F) and the total mitochondrial area (Figure 1G) were significantly higher in shDrp1 cells than in the control. The difference in total inside mitochondrial length was not significant (Figure 1E). Stable depletion of Drp1 resulted in a change in mitochondrial morphology, forming mitochondrial clusters at the perinuclear region (Figure 1C (clusters)). To quantify the formation of mitochondrial clusters, we measured the distance of each mitochondrion to its closest neighbor. We showed that the average mitochondrial distance was significantly smaller in shDrp1 cells than in control cells. We showed that the average mitochondrial distance was significantly smaller in shDrp1 cells than in control cells (Figure 1H).

**FIGURE 2:**
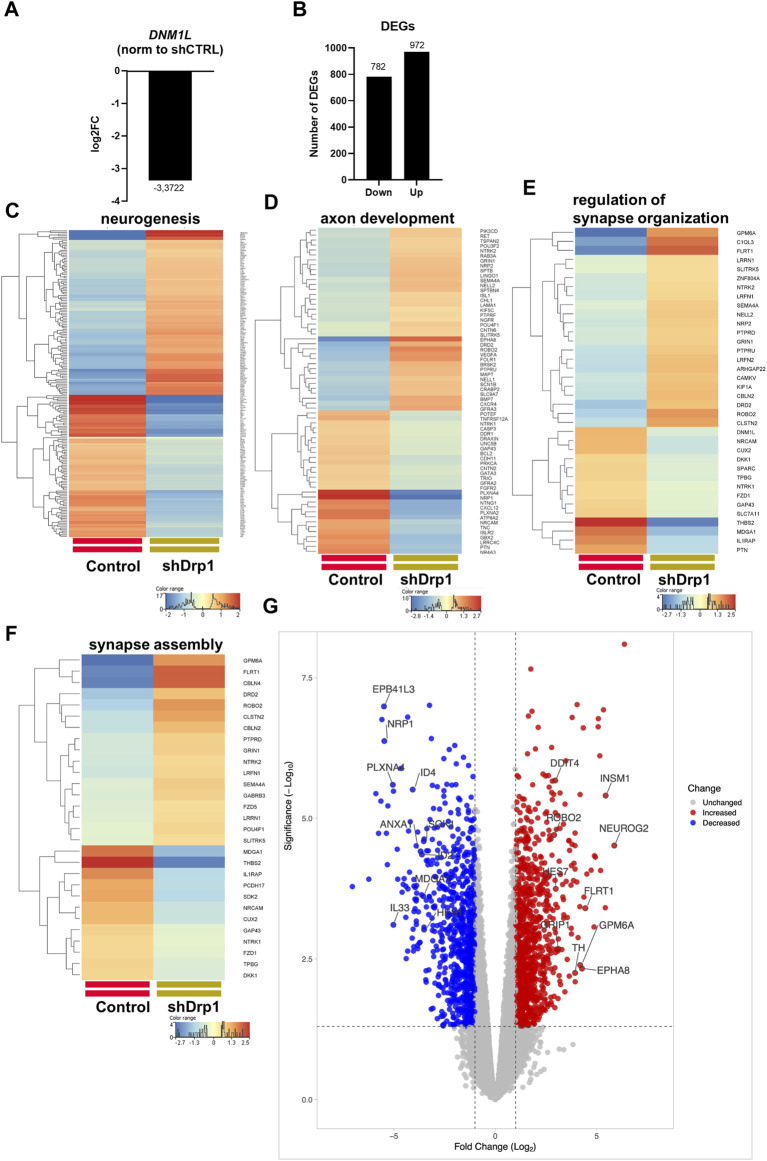
Drp1 deficiency affects the transcription of functionally relevant genes. **(A)** RNA-Seq data analysis from three independent experiments confirmed the depletion of DNM1L (gene name for Drp1) mRNA in the shDrp1 knockdown SH-SY5Y cells. **(B)** Compared to control cells, the differentially expressed genes (DEGs) in shDrp1 cells included 972 upregulated and 782 downregulated genes. Only significant DEGs are presented (Bejamini-Hochberg FDR corrected *p*-value <0.05). GO analysis of differentially expressed genes between shDrp1 and control cells significantly enriched several nerve system-related pathways (Bonferroni step-down corrected *p*-value <0.05). Heatmaps represent the expression patterns of genes of neurogenesis GO:0022008 **(C)**, axon development GO:0007409 **(D)**, regulation of synapse organization GO:0050807 **(E),** and synapse assembly GO:0007416 **(F)**. Heatmaps were generated by a hierarchical clustering algorithm using Ward’s linkage method, and similarity measures were determined by Euclidean distance metric with the StranNGS software. The color range shows the normalized gene expression values on the log2 scale; the histogram represents the distribution of the genes by their normalized values. **(G)** The Volcano plot was generated by the online application VolcaNoseR2.https://huygens.science.uva.nl/VolcaNoseR2/. The ten most significantly upregulated and downregulated genes of shDrp1 cells are shown.

The link between Drp1 and human diseases is the subject of increasing studies (Guo et al., 2013; Tanwar et al., 2016; Qi et al., 2019; Jin et al., 2021). To determine the effect of Drp1 on global gene expression, we performed mRNA sequencing and compared the transcriptomes of shDrp1 and control cells. DNM1L mRNA was substantially reduced in the SH-SY5Y knockdown cells (Figure 2A). In addition, Drp1 deficiency resulted in changes in gene expression. Of the differentially expressed genes (DEGs), 782 genes were significantly downregulated, and 972 genes were significantly upregulated in cells constitutively depleted of Drp1 compared to control cells (Figure 2B, Supplementary Table S5).

We performed Gene Ontology (GO) term enrichment analysis using the significantly upregulated and downregulated genes to find the significant biological relevance of the DEGs (Supplementary Tables S6, S7). GO analysis of DEGs revealed upregulation of cellular processes (Supplementary Figure S1). Heatmaps represent the expression patterns of genes related to neurogenesis, including regulation of neuron differentiation, generation of neurons, neuron differentiation, and neuron development (Figure 2C), axon development (Figure 2D), regulation of synapse organization (Figure 2E) and synapse assembly (Figure 2F) to be associated with Drp1 suppression. The ten most significantly upregulated genes of shDrp1 cells are related to the regulation of cell growth and cellular energy (DDIT4-DNA damage-inducible transcript 4 protein), neuronal differentiation (NEUROG2-neurogenin 2), navigation and projection of axons during neuronal development (ROBO2-Roundabout homolog 2), transcriptional regulation in neurogenesis (INSM1-Insulinoma-associated protein 1 and HES7-Transcription factor HES-7), promoting an increase both in neurite number and in neurite length (FLRT1-Leucine-rich repeat transmembrane protein), scaffolding and trafficking multiprotein signaling complexes in neurons (GRIP1-Glutamate receptor-interacting protein 1), neuronal differentiation and plasticity (GPM6A-Neuronal membrane glycoprotein M6-a), the biosynthesis of catecholamines, dopamine, noradrenaline, and adrenaline (TH-Tyrosine 3-monooxygenase), and axon guidance and neurite outgrowth (EPHA8-Ephrin type-A receptor 8) (Figure 2G and Supplementary Tables S5, S6). The ten most significantly downregulated genes are involved in the development of the cardiovascular system (NRP1- Neuropilin-1), apoptosis and angiogenesis (ID2- DNA-binding protein inhibitor ID-2, ID4- DNA-binding protein inhibitor ID-4), cytoskeleton remodeling (PLXNA4-Plexin A4), immune responses (ANXA1-Annexin A1, IL33-Interleukin 33), responses to environmental stress (SOK1- Serine/threonine-protein kinase 25) and DNA damage (HES1- Transcription factor HES-1), and the formation or maintenance of inhibitory synapses (MDGA1 MAM domain-containing glycosylphosphatidylinositol anchor protein 1) (Figure 2G and Supplementary Tables S5, S7). These findings suggest that Drp1 is an upstream regulator of essential cellular processes through transcriptional programming.

### shDrp1 cells show improved RA-BDNF-induced neuronal differentiation *in vitro*

**FIGURE 3:**
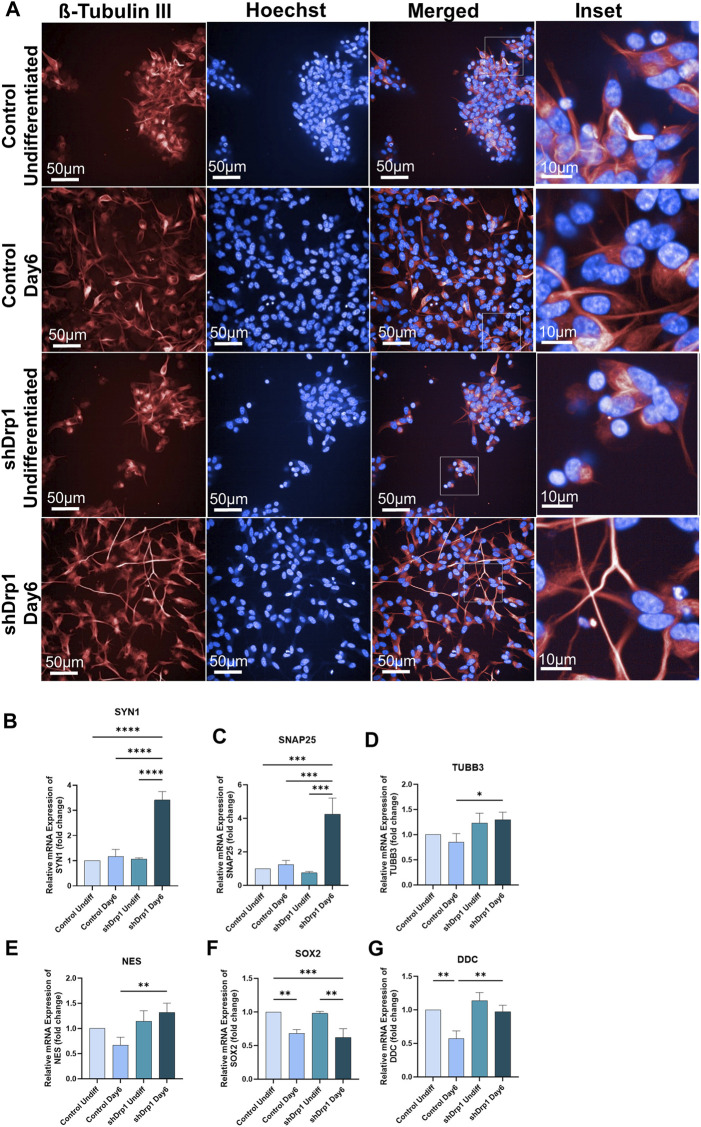
shDrp1 cells show increased neuronal differentiation with longer neurite outgrowth and more interconnected neuronal networks. Control and shDrp1 cells were treated with 10 μM RA on day 0 for 3 days (days 0–3) and 50 ng/mL BDNF on day 3 for 3 days (days 3–6) to induce neuronal differentiation. Automated live-cell confocal microscopy was performed on an Opera Phenix High Content Analysis system. A ×40 water objective was used for image acquisition settings. **(A)** Representative images of differentiated (Day 6) and undifferentiated control and shDrp1 live cells stained with Hoechst 33342 for nuclei and tubulin tracker for neuron-specific β-tubulin III. Images were merged to visualize both stains. Inset images reveal neurite outgrowth and the presence of several branches in differentiated cells. (Scale bar: 50 µm) **(B–G)** The mRNA levels of marker genes for neuronal differentiation, including SYN1 (Synapsin 1) **(B)**, SNAP25 (Synaptosomal-associated protein 25) **(C)**, TUBB3 (Tubulin beta-3 chain) **(D)**, NES (Nestin) **(E)**, SOX2 (Transcription factor SOX-2) **(F)** and DDC (Aromatic-L-amino-acid decarboxylase) **(G)** were measured by qPCR and using the 2^−ΔΔCT^ method in undifferentiated and differentiated control and shDrp1 cells. Data are presented as the mean ± SD of n ≥ 3 separate experiments. Statistical analysis was performed by ordinary One-way ANOVA with multiple comparisons (* indicates *p* < 0.05, ** indicates *p* < 0.01, *** indicates *p* < 0.001, **** indicates *p* < 0.0001).

To understand the role of Drp1 on neuronal cell differentiation, we differentiated Drp1 knockdown and control SH-SY5Y cells. The SH-SY5Y cell line is immortalized, highly proliferative, and widely used in neuroscience research (Kovalevich and Langford, 2013; de Medeiros et al., 2019; Ioghen et al., 2023). The cells were differentiated toward the neuronal lineage using an established protocol by Forster et al., 2016. The 2 cell lines were compared after completing phase 1 (days 0–3) and phase 2 (days 3–6) differentiation. Neuronal differentiation was monitored over time by bright-field and fluorescence microscopy on Days 3 and 6 (Supplementary Figure S2). Live cell imaging with tubulin tracker staining (Figure 3A) and neuron-specific β-III tubulin (Tuj1) staining of fixed cells (Supplementary Figure S3) confirmed neuronal differentiation. Undifferentiated (Day 0) control and shDrp1 cells show clumping (Figure 3A). The differentiated cells clearly show morphological characteristics of neurons, including neurofilament outgrowth and an interconnected neuronal network. Our imaging demonstrates that shDrp1 cells had longer neurite outgrowth and more interconnected neuronal networks than differentiated control cells, indicating a greater extent of neuronal differentiation in the shDrp1 cells (Figures 3A, insets).

qRT-PCR was performed on selected neuronal markers, and the mRNA fold change was calculated using the 2^−ΔΔCT^ method in undifferentiated (Day 0) and differentiated (Day 6) cell lines. According to Forster et al., 2016, RA-BDNF-induced neuronal differentiation of SH-SY5Y cells does not represent a fully mature neurotransmitter signature. When we compared the undifferentiated control cells to the differentiated control cells, synaptic markers, such as SYN1 (Synapsin1) and SNAP25 (Synaptosomal-associated protein 25), showed an increasing but not significant trend in the differentiated control cells (Figures 3B,C). The expression of NES (the gene that encodes Nestin, which is required for central nervous system development) (Yaworsky and Kappen, 1999) showed no significant differential expression in differentiated *versus* undifferentiated control cells (Figure 3E), similar to the result obtained by Forster et al., 2016. SOX2, a transcription factor and a marker of pluripotency, decreased significantly in control cells, indicating a commitment towards differentiation (Figure 3F). On the other hand, the gene expression of SYN1, SNAP25, and the axonal marker TUBB3 (Tubulin beta-3) increased and SOX2 decreased significantly in differentiated shDrp1 cells compared to undifferentiated shDrp1 cells (Figures 3B–D,F). Comparing the gene expression in differentiated shDrp1 to differentiated control cells, DDC (Dopa decarboxylase; a serotonergic marker) expression was significantly higher in differentiated shDrp1 cells (Figure 3G). In addition, we found a significant increase in the gene expression of SYN1, SNAP25, TUBB3, and NES in differentiated shDrp1 compared to differentiated control (Figures 3B–D).

**FIGURE 4:**
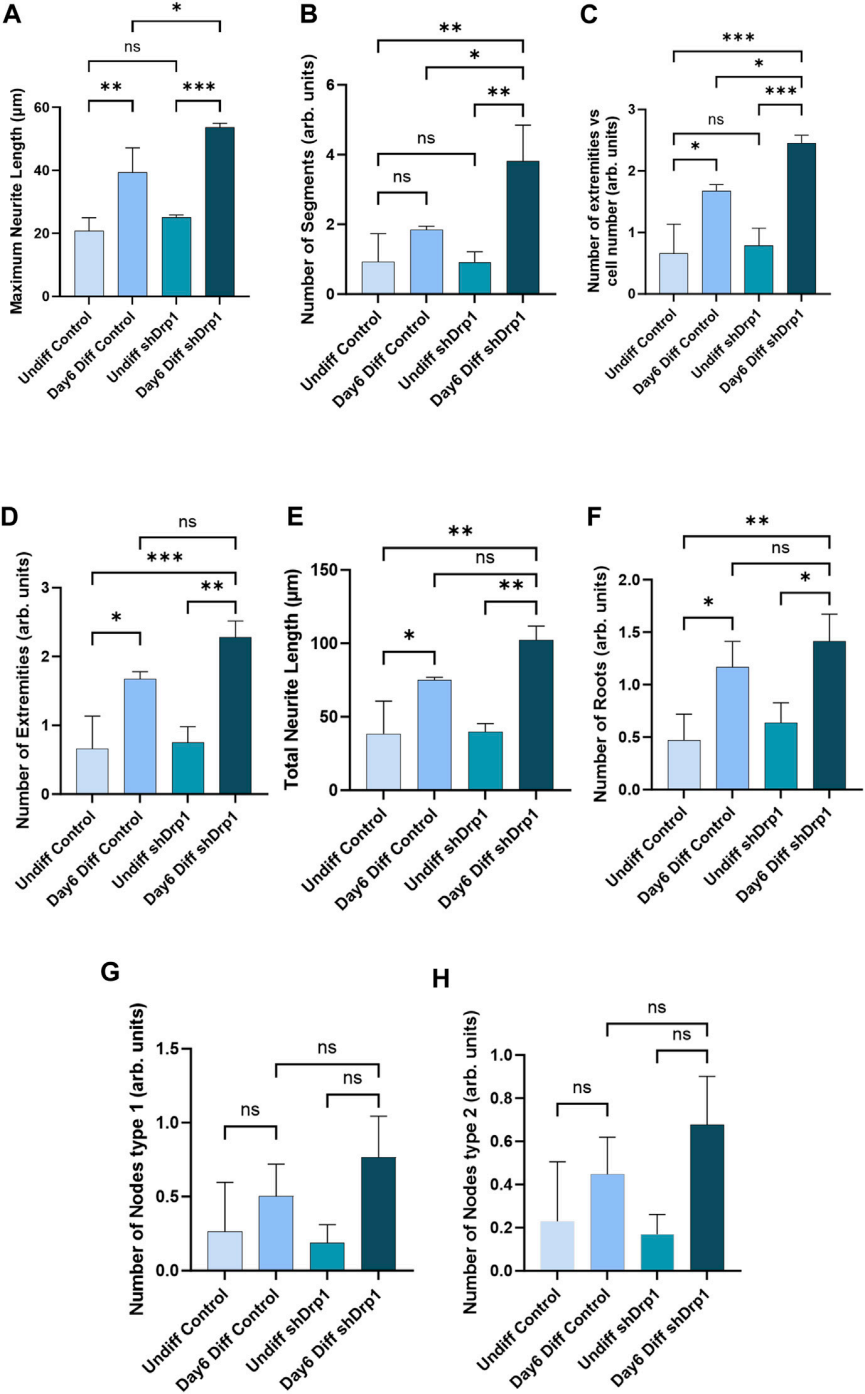
Quantitative analysis demonstrates a significant increase in neurite outgrowth parameters in shDrp1 cells. Automated confocal microscopy analysis of neurite outgrowth in undifferentiated and differentiated (Day 6) control and shDrp1 live cells was performed on an Opera Phenix High Content Analysis system. Live cells were stained with Hoechst 33342 for nuclei and tubulin tracker for neuron-specific β-tubulin III. Quantitative analysis was performed using the built-in “CSIRO neurite Analysis 2” method of the Harmony 4.9 software. **(A–H)** Defined parameters were analyzed in three independent experiments. Data are presented as the mean ± SD of three independent experiments (*n* = 3). 39677 differentiated and 39658 undifferentiated shDrp1 cells and 45066 differentiated and 22568 undifferentiated control cells were analyzed. Groups were compared using ordinary One-way ANOVA with multiple comparisons (* indicates *p* < 0.05, ** indicates *p* < 0.01, *** indicates *p* < 0.001, ns indicates not-significant).

To quantitatively assess the morphology of neuronal differentiation, we performed an automated analysis of neurite outgrowth and branching of our differentiated cells using the CSIRO neurite outgrowth analysis algorithm incorporated into the High Content Analysis System (Opera™, PerkinElmer) (Figure 4). Quantitative morphological live-cell assessment including multiple parameters such as maximum neurite length (Figure 4A), number of extremities (Figures 4C,D), total neurite length (Figure 4E), and number of roots (Figure 4F) significantly increased in both differentiated shDrp1 and control cells compared to undifferentiated shDrp1 and control, respectively. We also observed a significant increase in the number of segments in differentiated shDrp1 cells compared to undifferentiated shDrp1 cells (Figure 4B). Measurement of neurite outgrowth is critical for assessing nervous system development. We found that the value of maximum neurite length of differentiated shDrp1 cells was significantly higher than the maximum length of differentiated control cells, indicating that differentiated shDrp1 cells have extended outgrowth to connect synaptically with other neuron-like cells (Figure 4A). The number of segments connecting branch points or neuron bodies, and the number of extremities (the total number of neurite segments with a terminal endpoint) per cell was also significantly higher in differentiated shDrp1 cells compared to differentiated control cells (Figures 4B,C). Other parameters, including node types, were higher, but the differences were not significant (Figures 4G,H). These results suggest that reduced expression of Drp1 enhances RA-BDNF-induced neuronal differentiation of SH-SY5Y cells *in vitro.* In summary, this effect may be caused by the widespread transcriptomic changes induced by the stable knockdown of Drp1.

### Reduced ERK1/2 activation in differentiated shDrp1 cells

**FIGURE 5:**
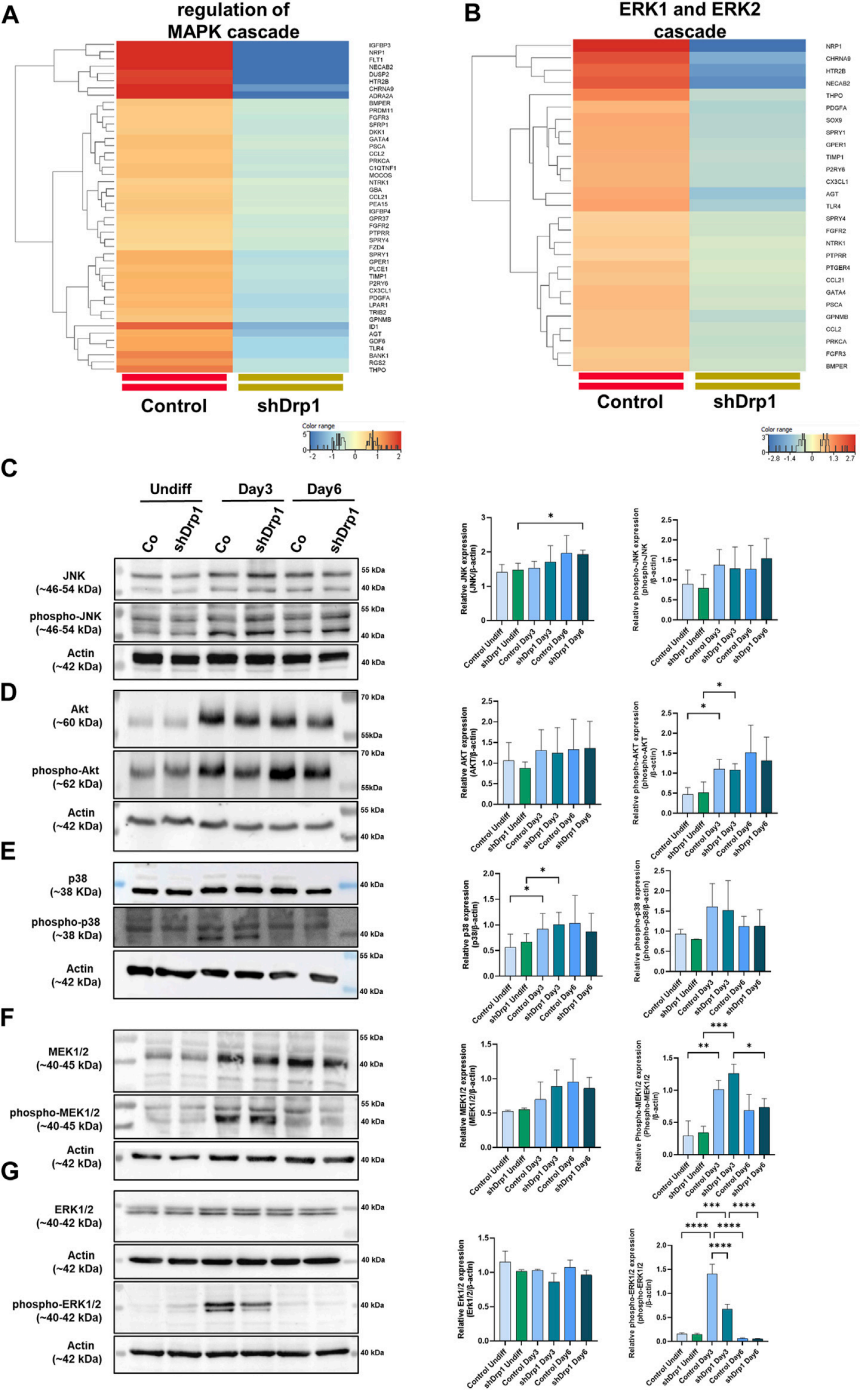
Reduced phosphorylation of ERK1/2 in shDrp1 cells on Days 3 after RA-induced differentiation. Heatmaps represent the expression patterns of genes regulating MAPK cascade GO:0043408 **(A)** and ERK1 and ERK2 **(B)** cascade GO:0070371. Heatmaps were generated by a hierarchical clustering algorithm using Ward’s linkage method, and similarity measures were determined by Euclidean distance metric with the StranNGS software. The color range shows the normalized gene expression values on the log2 scale, and the histogram represents the distribution of the genes by their normalized values. The protein- and phosphoprotein levels of members of cellular signaling pathways, including JNK, phospho-JNK **(C)**, Akt, phospho-Akt **(D)**, p38, phospho-p38 **(E)**, MEK1/2, phospho-MEK1/2 **(F)** and ERK1/2, phospho-ERK1/2 **(G)** were examined by western blotting in undifferentiated and differentiated (Days 3 and 6) control and shDrp1 cells. Cells were untreated or treated with 10 μM RA for 3 days (0–3) and 50 ng/mL BDNF for the next 3 days (3–6) to achieve neuronal differentiation. Cells were lysed with RIPA buffer, and an equal amount of protein was separated. β-actin was used as an internal loading control. Representative images of western blots are shown. Statistical analyses of the relative protein expressions are shown. Images were taken using a ChemiDoc Imager, and the pixel intensity was quantified and normalized to the internal control, β-actin, using Image Lab software. Data are presented as means ± SD (*n* = 4). Groups were compared using One-way ANOVA with multiple comparisons. Only significant differences are shown (* indicates *p* < 0.05, ** indicates *p* < 0.01, *** indicates *p* < 0.001, **** indicates *p* < 0.0001).

According to our transcriptomic data, the knockdown of Drp1 modulates the expression of genes involved in protein phosphorylation (Supplementary Figure S4, Supplementary Table S5). Moreover, GO terms for genes that regulate the MAPK and ERK1/2 signaling pathways were significantly downregulated after the Drp1 knockdown in undifferentiated cells (Figures 5A,B, Supplementary Table S7). Emerging studies suggest that RA-BDNF-induced differentiation activates different signaling pathways with diverse roles during neuronal differentiation. Thus, the activation and phosphorylation status of signaling molecules after RA-BDNF-induced neuronal differentiation in control and Drp1 knockdown cells were assessed by immunoblot analysis of undifferentiated and Days 3 and 6 differentiated cells. JNK and Akt phosphorylation were increased on Day 3 in both cell lines, with a significant change for phospho-Akt. Both JNK and Akt remained phosphorylated on Day 6 in both cell lines, indicating that RA induces the phosphorylation of JNK and Akt, and their activation is required for phase 2 differentiation (Figures 5C,D). RA-induced phosphorylation of p38, MEK1/2, and ERK1/2 was elevated on Day 3 in control, indicating the activation of the ERK-MAPK pathway by RA during the early differentiation phase (Figures 5E–G).

Interestingly, phosphorylated ERK1/2 was significantly lower on Day 3 in shDrp1 cells compared to the control cells (Figure 5G), and according to our western blot, only ERK1 was weakly phosphorylated. This result suggests that the suppression of Drp1 might result in ERK1/2 independent neuronal differentiation in SH-SY5Y cells. By Day 6, phosphorylation of p38, MEK1/2, and ERK1/2 decreased to the levels of undifferentiated cells (Figures 5E–G), suggesting that BDNF does not induce the phosphorylation of p38, MEK1/2, and ERK1/2 in SH-SY5Y cells. In summary, these results indicate that downregulation of Drp1 modulates the activation of ERK1/2 during RA-induced neuronal differentiation.

### ERK1/2 dephosphorylation is independent of DUSP1 and DUSP6 in shDrp1 cells during neuronal differentiation

**FIGURE 6:**
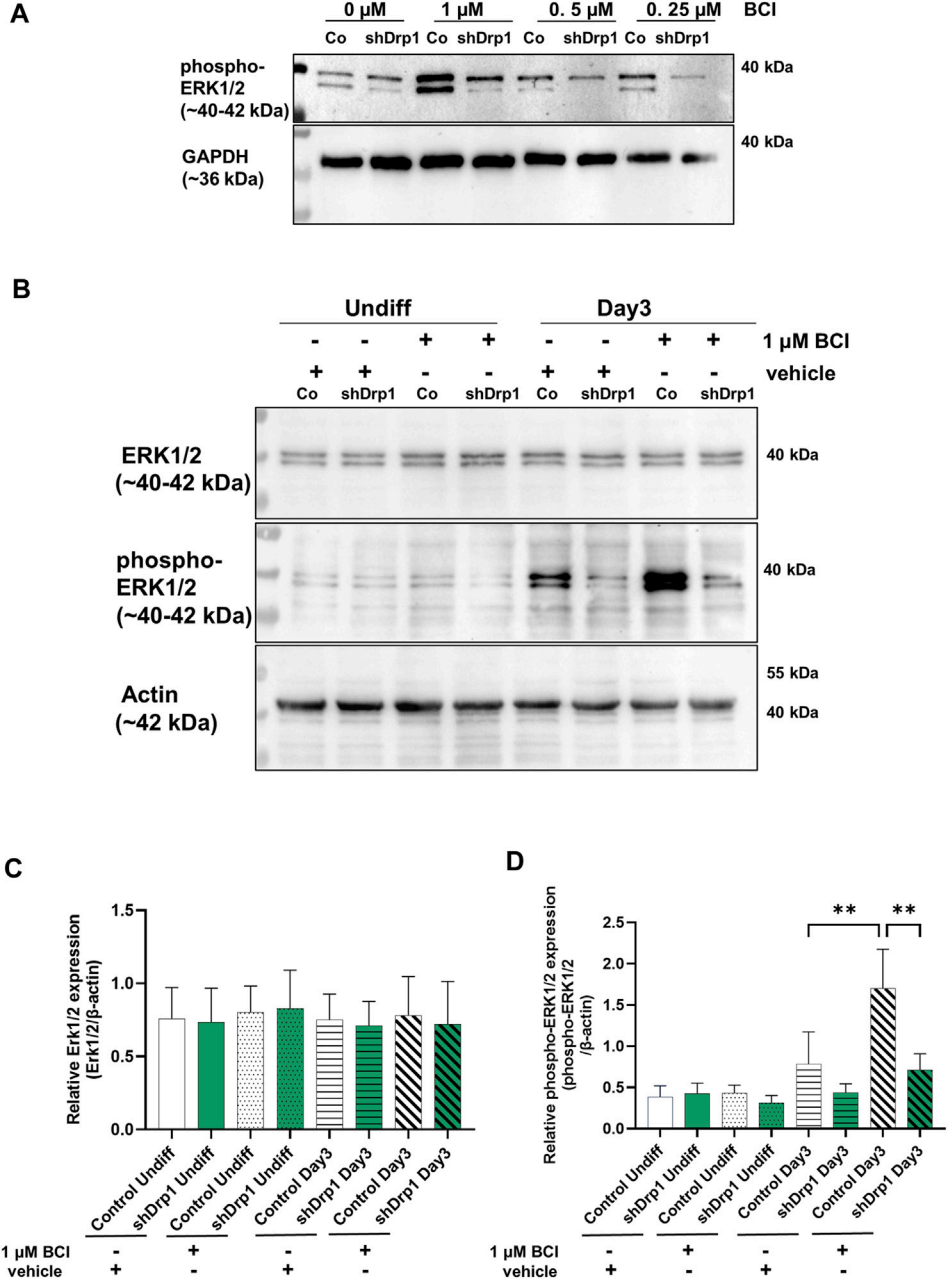
Inhibition of dual specificity phosphatases 1 and 6 does not lead to increased phospho-ERK1/2 in shDrp1 cells. **(A)** The optimal working concentration of BCI was obtained by the following: Cells were treated with 10 μM RA for 3 days to induce phase 1 differentiation. Control and shDrp1 cells were treated with 0.1 vol% vehicle DMSO or different concentrations of BCI for 24 h on Day 2. Cells were lysed with RIPA buffer, and an equal amount of proteins were separated to examine the level of phospho-ERK1/2. GAPDH was used as the internal loading control. **(B)** The protein and phosphoprotein levels of ERK1/2 were examined by SDS-PAGE and western blotting in undifferentiated and differentiated (Day 3) control and shDrp1 cells. Cells were treated with DMSO or 10 μM RA for 3 days to induce phase 1 differentiation. Control and shDrp1 cells were treated with vehicle or 1 μM BCI for 24 h on Day 2. Cells were lysed with RIPA buffer, and an equal amount of proteins were separated. β-actin was used as the internal loading control. Representative images of western blots are shown. Statistical analyses of the relative protein expression of ERK1/2 **(C)** and phospho-ERK1/2 **(D)** are shown. Images were taken with a ChemiDoc Imager, and the pixel intensity was quantified and normalized to the internal control β-actin using Image Lab software. Data are presented as means values ±SD (*n* = 4). Groups were compared using ordinary One-way ANOVA with multiple comparisons. Only significant differences are shown (** indicates *p* < 0.01).

The phosphorylation of ERK1 and ERK2 is catalyzed by the dual-specificity mitogen-activated protein kinases 1 and 2 (MEK1/2). MEK1/2 was similarly phosphorylated and activated during RA-induced differentiation until Day 3 in both cell lines. Therefore, the differences in the phosphorylation level of ERK1/2 may be due to the phosphatases that dephosphorylate ERK1/2. ERK1/2 is dephosphorylated by the dual specificity phosphatases 1 and 6 (DUSP1 and DUSP6). Next, we assessed the effects of the DUSP1 and DUSP6 inhibitor BCI on phospho-ERK1/2 levels during differentiation in control and shDrp1 cells. Cells were induced with RA on Day 0, and on Day 2, the indicated concentrations of BCI were added for 24 h to determine the optimal experimental concentration of BCI. Cells were collected on Day 3 after completing phase 1 differentiation. 1 μM BCI treatment caused robust phosphorylation of ERK1/2 in control but not shDrp1 cells (Figure 6A). Thus, we used 1 μM BCI treatment for further experiments. ERK1/2 protein level and phosphorylation were compared in undifferentiated and differentiated control and shDrp1 cells with or without 1 μM BCI treatment. BCI did not cause changes in the ERK1/2 level in control and shDrp1 cells (Figures 6B,C) and did not induce elevated phospho-ERK1/2 levels in undifferentiated control or shDrp1 cells (Figures 6B,D). RA significantly increased phospho-ERK1/2 levels in Day 3 differentiated control cells compared with the phospho-ERK1/2 levels in undifferentiated control cells. Furthermore, BCI treatment significantly increased phosphorylated ERK1/2 in Day 3 differentiated control cells (Figures 6B,D). Remarkably, BCI treatment did not affect phospho-ERK1/2 levels in differentiated shDrp1 cells, suggesting that dephosphorylation of ERK1/2 is independent of DUSP1 and 6 in shDrp1 cells.

### Mitochondrial network rearrangement and metabolic adaptation of control and shDrp1 cells during neuronal differentiation

Drp1 is a crucial regulator of mitochondrial dynamics. The shape and morphology of mitochondria greatly depend on their function. The morphology of mitochondria changes during the differentiation of neural stem cells in the developing and adult brain and is regulated by mitochondrial fusion and fission proteins. Mitochondria of differentiated neurons in the developing brain show an elongated structure. In the adult hippocampus, mitochondria show a highly elongated morphology (Khacho et al., 2016; Beckervordersandforth et al., 2017; Khacho and Slack, 2018).

**FIGURE 7:**
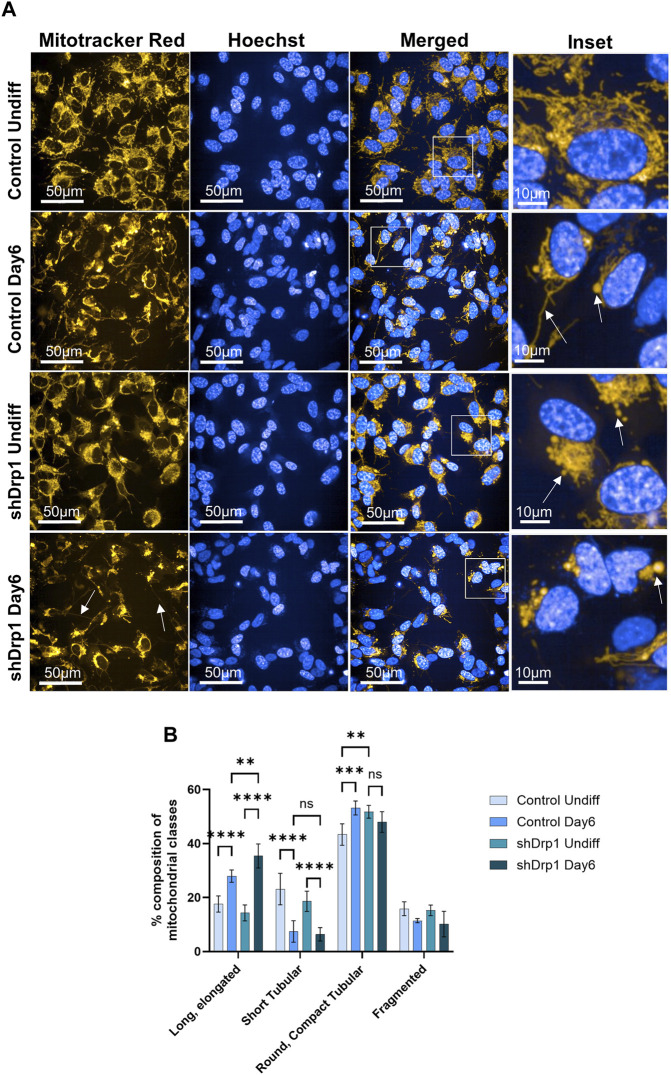
Mitochondrial network remodeling of control and shDrp1 cells during neuronal differentiation. Cells were treated with 10 μM RA for 3 days (0–3) and 50 ng/mL BDNF for the next 3 days (3–6) to achieve neuronal differentiation. **(A)** Automated confocal microscopy was performed on an Opera Phenix High Content Analysis system. A ×63 water objective was used for image acquisition settings. Representative images for mitochondrial morphology in undifferentiated and differentiated (Day 6) control and shDrp1 live cells stained with MitoTracker Red CMXRos for mitochondria and Hoechst 33342 for nuclei are shown**. (B)** Quantification of mitochondrial classes was performed with the built-in Harmony 4.9 and PhenoLogic machine-learning software. Mitochondria were classified as long, elongated tubular, short tubular, round, compact tubular, and fragmented. The number of objects (mitochondrial populations) were counted in undifferentiated and differentiated control and shDrp1 cells and were expressed as percent % of the overall. > 300 cells were analyzed/sample. Statistical analysis was performed by two-way ANOVA with multiple comparisons (** indicates *p* < 0.01, *** indicates *p* < 0.001, **** indicates *p* < 0.0001, and ns indicates not-significant).

A recent study confirmed that differentiating SH-SY5Y cells exhibited a significant increase in cells with tubular mitochondria (D'Aloia et al., 2024). Thus, the effects of reduced Drp1 levels on mitochondrial morphology during neuronal differentiation of SH-SY5Y cells were investigated. We assessed mitochondrial morphology using live cell high-content imaging analysis with Mitotracker Red CMXRos to stain mitochondria and Hoechst to stain nuclei. On Day 6, control cells exhibited interconnected tubules with elongated structures and compact “pearl-like” tubules (marked with arrows on Figure 7A insets) compared to the separate tubules of undifferentiated control, suggesting remodeling (Figure 7A upper panels and insets). The mitochondrial networks of shDrp1 cells remained highly fused with asymmetric, typically perinuclear distribution with “pearl-like” and elongated interconnected tubules (marked with arrows on Figure 7A insets), suggesting that mitochondria of shDrp1 cells retained the hyperfused mitochondrial structure but also developed long, elongated, highly interconnected tubules during differentiation, a result similar to that obtained by Vantaggiato et al., 2019 in mouse embryonic carcinoma P19 cells (Figure 7A lower panels and insets). To further corroborate the microscopic data, we quantified mitochondrial phenotypes of undifferentiated and differentiated control and shDrp1 cells using live-cell high-content image analysis with a method established earlier by our group (Douida et al., 2021). Quantification confirmed a significantly increasing extended, elongated, and round, compact tubular mitochondrial populations in both cell lines during differentiation. Furthermore, mitochondria seem to go through a morphological change from short to long tubular structure. Differentiated shDrp1 cells represent significantly higher populations of extended, elongated phenotype and round, compact tubular mitochondria than differentiated control (Figure 7B).

**FIGURE 8:**
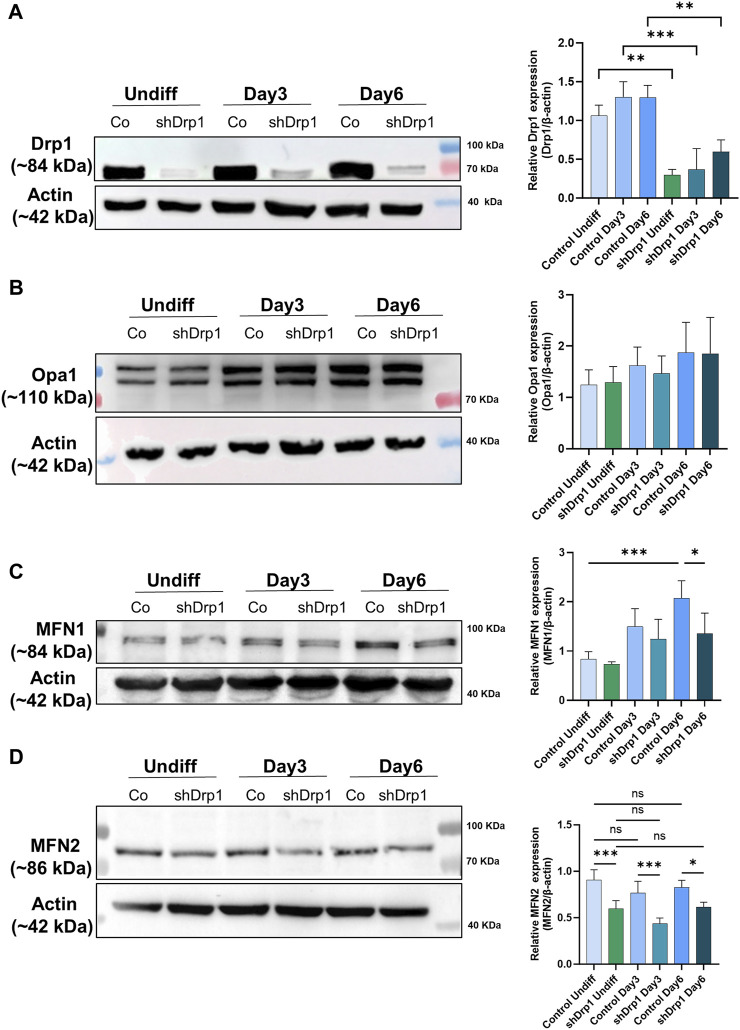
Reduced expression of mitochondrial fusion proteins MFN1 and MFN2 in shDrp1 cells compared to control cells. The protein levels of mitochondrial fission and fusion proteins were examined using anti-DNM1L **(A)**, anti-Opa1 **(B)**, anti-MFN1 **(C)**, and anti-MFN2 **(D)** antibodies in undifferentiated and differentiated (Days 3 and 6) control and shDrp1 cells. Cells were lysed with RIPA buffer, and an equal amount of protein was separated using SDS-PAGE and analyzed by western blotting. β-actin was used as an internal loading control. Representative images of western blots are shown. Statistical analyses of the relative protein levels are shown. Images were taken using a ChemiDoc Imager, and the pixel intensity was quantified and normalized to the internal control, β-actin, using Image Lab software. Data are presented as mean values ±SD (n ≥ 3). Groups were compared using One-way ANOVA with multiple comparisons (* indicates *p* < 0.05, ** indicates *p* < 0.01, *** indicates *p* < 0.001, and ns indicates not-significant).

Drp1 protein levels increased slightly during differentiation in control cells, but the difference was not significant. The constitutive depletion of Drp1 was maintained in shDrp1 cells during RA-BDNF-induced differentiation (Figure 8A). We also evaluated the phosphorylation of Drp1 at Ser-637 and Ser-616 in control cells during neuronal differentiation. RA induced the phosphorylation of Drp1 at Ser-637 by Day 3, and the phosphorylation disappeared by Day 6. The phosphorylation of Drp1 at Ser-616 remained steady during differentiation (Supplementary Figure S5). The mitochondrial fusion protein Opa1 level showed an elevated but not significant trend in both cell lines during differentiation (Figure 8B). MFN1 increased significantly during differentiation in control, not in shDrp1 cells (Figure 8C), and MFN2 levels did not change significantly in any of the cell lines during differentiation (Figure 8D). MFN1 protein levels were lower (significant at Day 6) and MFN2 levels were markedly lower in shDrp1 cells compared to the respective controls during differentiation (Figures 8C,D). These results are consistent with the data obtained in *Drp1* ^*−/−*^ MEF by Wakabayashi et al., 2009, suggesting that mitochondrial fusion may compensate for the loss/reduction of a critical fission protein independent of cell type.

**FIGURE 9:**
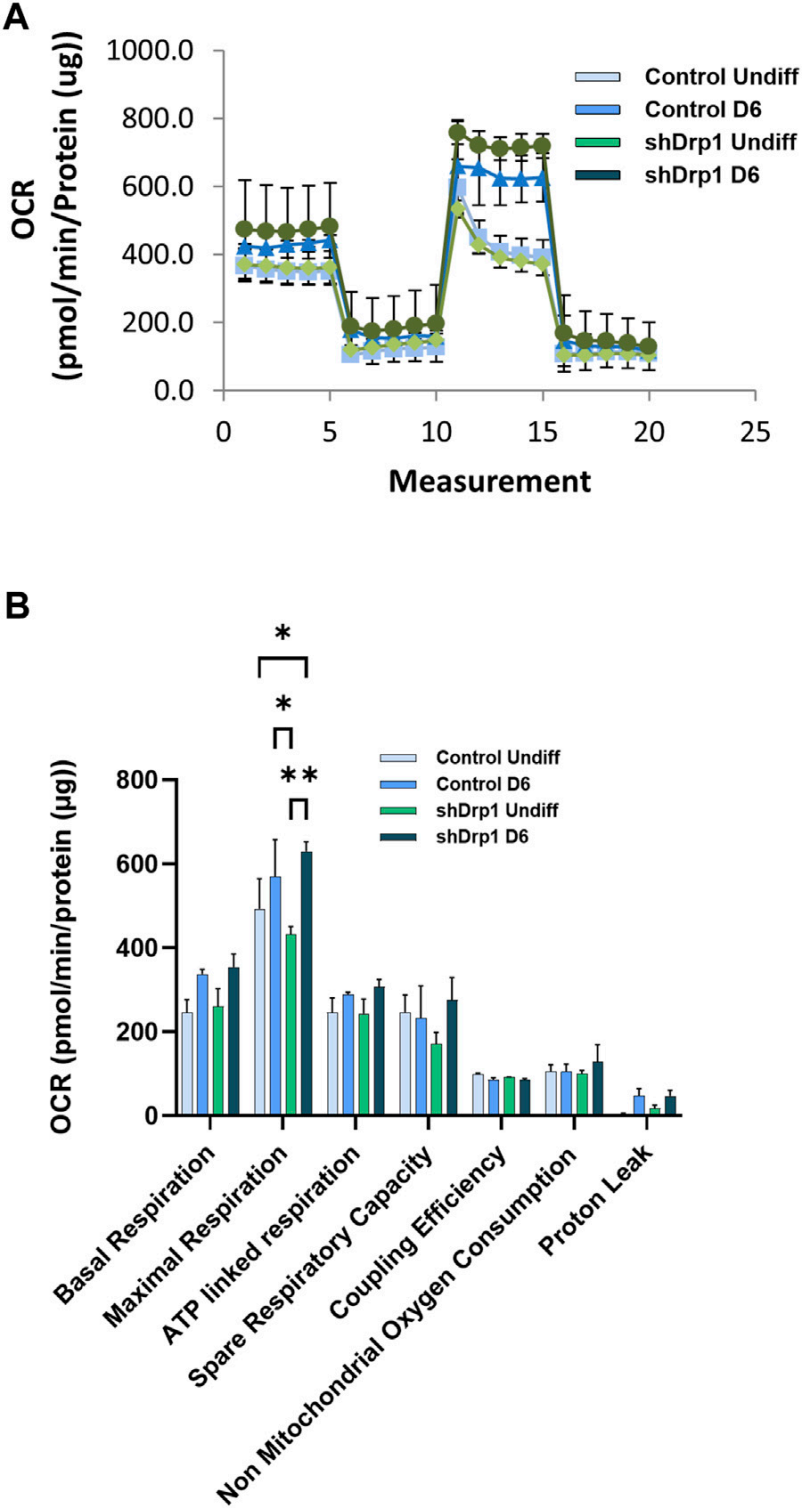
Increased maximal respiration in differentiated shDrp1 cells. Oxygen consumption rate (OCR) was measured in undifferentiated Day 6 differentiated control and shDrp1 cells. **(A)** The basal OCR was determined 30 min before and after mitochondrial sequential addition of 1.5 µM oligomycin (olig), 1 µM FCCP, and 1 µM rotenone/antimycin A cocktail (Anti/Rot). **(B)** Calculated parameters of mitochondrial respiration based on measured OCR. Data were normalized to total protein (pmol/min/µg protein). Data were analyzed using Wave Desktop software. Data are presented as means ± SEM of *n* = 3 independent experiments. Statistical analysis was performed by two-way ANOVA with multiple comparisons. Only significant differences are shown (* indicates *p* < 0.05, ** indicates *p* < 0.01).

We also analyzed mitochondrial bioenergetics during differentiation because mitochondrial dynamics and bioenergetics closely influence each other. Oxygen consumption rate (OCR) was measured in real-time in undifferentiated and Day 6 differentiated cells in both control and shDrp1 cells. Basal OCR and OCRs were measured after adding selective mitochondrial inhibitors in the following sequential order: oligomycin, cyanide-p-trifluoromethoxyphenylhydrazone (FCCP), and a combination of rotenone and antimycin A. Oligomycin prevents protons from entering the mitochondria by binding to and inhibiting ATP synthase. Uncoupled mitochondrial respiration (maximal respiration) was measured by adding FCCP, which uncouples mitochondrial respiration and reduces ATP synthesis by collapsing the proton gradient across the inner mitochondrial membrane. The complex I inhibitor rotenone and the complex III inhibitor antimycin A were used to inhibit mitochondrial respiration to distinguish between mitochondrial and non-mitochondrial oxygen consumption (Figure 9A). Data from independent biological replicates were processed and analyzed by Wave desktop and Multi-File XF report generator. Seahorse analysis highlighted that undifferentiated shDrp1 cells did not differ from undifferentiated control in terms of mitochondrial bioenergetics. That suggests that stable knockdown of Drp1 modulates mitochondrial morphology but not bioenergetics at the undifferentiated state. A significant increase in maximal respiration was observed in differentiated shDrp1 cells compared to undifferentiated shDrp1 and differentiated control. An increasing but not significant trend emerged in basal respiration, ATP-linked respiration, proton leak, and spare respiratory capacity in differentiated control and shDrp1 cells (Figure 9B).

### Drp1 is dispensable for cell death after exposure to RA and mitochondrial complex inhibitors

**FIGURE 10:**
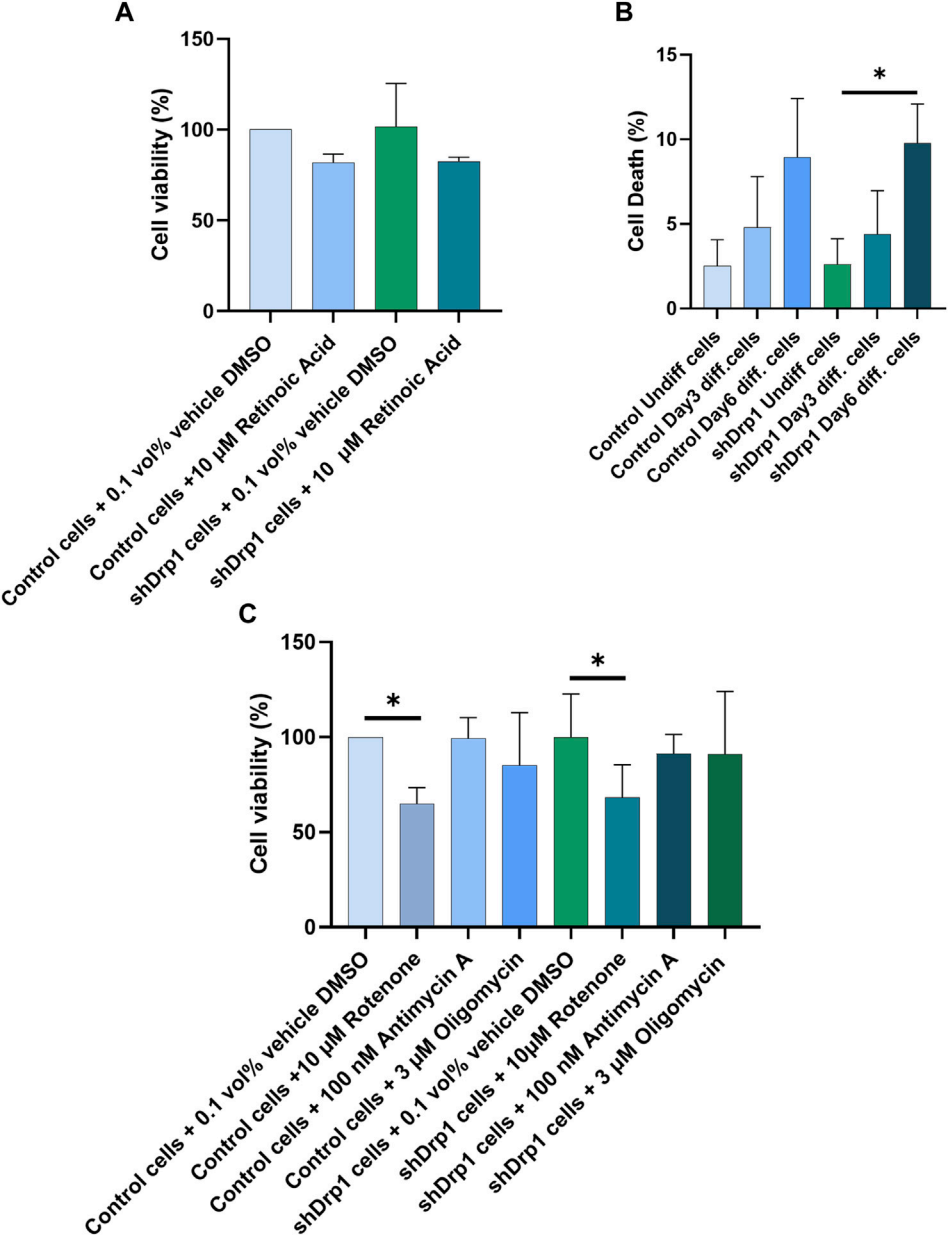
Retinoic acid and selective mitochondrial inhibitors induce cell death in Drp1-depleted cells to a similar extent than in control. **(A)** Cell viability was assessed using the sulphorhodamine B (SRB) assay. Undifferentiated control and shDrp1 cells were treated with 0.1 vol% vehicle DMSO and 10 µM RA. Data are presented as the means ± SD of three independent experiments. Groups were compared using One-way ANOVA with multiple comparisons. **(B)** The effects of Drp1 depletion on cellular death after differentiation. Cellular death was monitored using propidium iodide (PI) staining in undifferentiated and differentiated control and shDrp1 cells. Results are presented as means ± SD of three independent experiments. Statistical analysis was performed using One-way ANOVA with multiple comparisons (* indicates *p* < 0.05). **(C)** Assessment of cell viability using the SRB assay. Undifferentiated control and shDrp1 cells were treated with DMSO, 10 µM Rotenone, 3 µM Oligomycin, or 100 nM Antimycin **(A)**. Data are presented as means ± SD of three independent experiments. Statistical analysis was performed using One-Way ANOVA with multiple comparisons. Only significant differences are shown (* indicates *p* < 0.05).

RA mediates neuronal differentiation (Park et al., 2016; Teppola et al., 2016; Dutta et al., 2023). In addition, RA inhibits cell growth and induces cell death and differentiation in various tissues (Wolf, 2008; Noy, 2010; Cheng et al., 2013; Kang and Koh, 2023). To test whether the constitutive depletion of Drp1 affects cell viability during RA-induced differentiation, we treated both control and shDrp1 cells with 10 μM RA or vehicle (DMSO) for 3 days, and cell viability was assessed using sulphorhodamine B (SRB) assays, which measure cellular protein content (Figure 10A). RA induced a non-significant decrease in the viability of both cell lines, and no differences were detected between control and shDrp1 cells. To confirm the results of the SRB assay, we performed differentiation and assessed cell death by propidium iodide staining, which excludes live and apoptotic cells and stains late-apoptotic and necrotic cells. A minimal increase in cell death at Days 3 and 6 in both cell lines was detected. Cell death increased significantly in shDrp1 cells on Day 6, but no significant differences were detected between control and shDrp1 cells (Figure 10B).

Drp1 is critical for mitochondrial fission to initiate cell death, providing cellular quality control when cells are exposed to stress stimuli such as mitochondrial toxins. We used the ATP synthase inhibitor oligomycin, the complex I inhibitor rotenone, and the complex III inhibitor antimycin A to monitor cell vulnerability to cell death in control and Drp1 knockdown cells according to our previously described method (Douida et al., 2020) using the SRB assay. Cell viability remained high after 100 nM Antimycin A treatment in both cell lines. Although 3 μM oligomycin and 10 μM rotenone treatment decreased cell viability in both cell lines, the differences were significant only for rotenone (Figure 10C). We concluded that Drp1 does not modulate RA-induced cell death in differentiated cells and mitochondrial inhibitor-induced toxicity in undifferentiated neuroblastoma cells *in vitro*.

### Downregulation of Drp1 causes late formation and smaller size of toxic huntingtin protein aggregates

**FIGURE 11:**
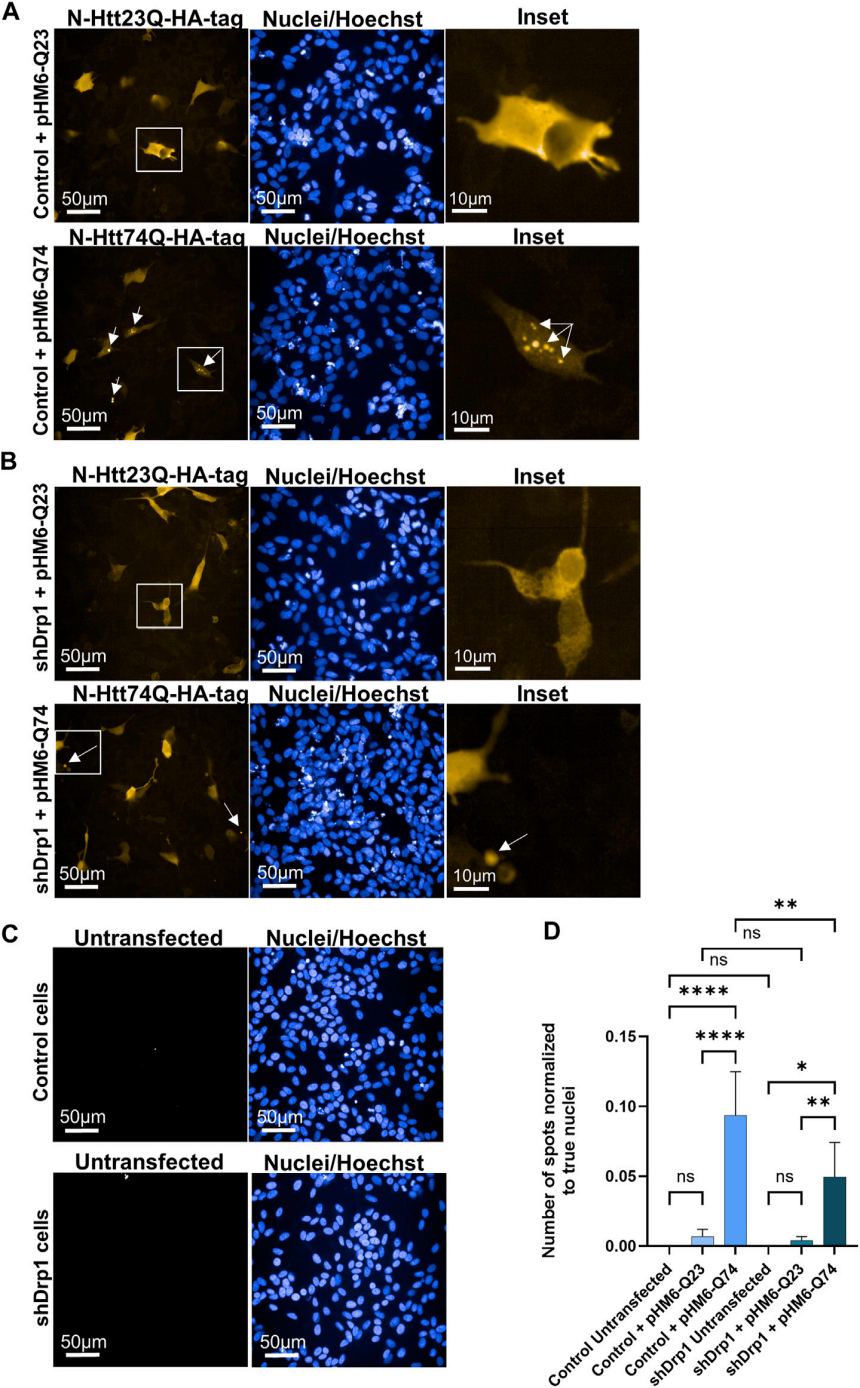
Stable knockdown of Drp1 delays the aggregate formation of mutant N-Htt fragments. Control and shDrp1 cells were transfected with pHM6-Q23 (wild type N-Htt) (**(A,B)** upper panels) and pHM6-Q74 (mutant N-Htt) (**(A,B)** lower panels) plasmids, and the overexpression of N-Htt fragments was measured after 72 h. Representative images show cells immunolabeled with anti-HA-tag/Alexa Fluor 568 for Q23 and Q74. Hoescht 33342 was used to stain the cell nuclei. Arrows on inset images indicate toxic N-Htt aggregates. **(C)** Untransfected cells were used for transfection control and immunolabeled as the transfected cells. **(D)** Quantitative analysis was performed using the “Spot Analysis” module of the Harmony 4.9 software. The insoluble mutant N-Htt fragments were segmented on the Alexa 568 channel. The numbers of shDrp1 analyzed cells were 23351 untransfected, 49701 pHM6-Q23, and 52920 pHM6-Q74 transfected cells. The numbers of control cells analyzed were as follows: 10945 untransfected, 39873 pHM6-Q23, and 43273 pHM6-Q74 transfected cells. Data are expressed as means ± SD of *n* = 5 independent experiments. Groups were compared using One-way ANOVA with multiple comparisons (* indicates *p* < 0.05, ** indicates *p* < 0.01, **** indicates *p* < 0.0001, and ns indicates not significant).

The mutant huntingtin protein abnormally interacts with Drp1 and stimulates the enzymatic activity of Drp1, leading to neuronal cell death in mice and humans with Huntington’s disease (Song et al., 2011; Shirendeb et al., 2012). Thus, the effects of Drp1 knockdown on the formation of toxic aggregates of the mutant huntingtin protein were investigated. Cells were transiently transfected to overexpress the N-Htt fragment with 23 polyQ repeats representing the normal length of the protein and the mutant (toxic) N-Htt fragment with 74 polyQ repeats in undifferentiated control and shDrp1 cells. Both inserts are fused to HA-tag for immunolabeling. Protein expression was monitored for 72 h after transfection in fixed cells using high-content analysis. Confocal microscopic image analysis revealed a cytoplasmic distribution of the N-Htt fragment with 23 polyQ in both cell lines (Figures 11A,B upper panels). Cells expressing the mutant N-Htt fragment displayed a mixed population of cells showing a cytoplasmic distribution and aggregates with different sizes and numbers or both of the mutant N-Htt (Figures 11A,B lower panels). To quantitatively assess toxic huntingtin aggregate formation, fluorescent intensity properties normalized to true cell nuclei using batch analysis in untransfected cells (Figure 11C), and cells expressing the normal N-Htt and the mutant N-Htt fragments were analyzed. The number of aggregates in control and shDrp1 cells expressing the mutant N-Htt fragments was significantly increased compared to the respective untransfected cells or cells expressing the normal length of the N-Htt fragment with 23 polyQ. Interestingly, toxic huntingtin aggregates were significantly reduced in Drp1 knockdown cells expressing the mutant N-Htt compared to control cells expressing the mutant N-Htt (Figure 11D). Collectively, these data suggest that the depletion of Drp1 reduces the formation of neurotoxic huntingtin aggregates in undifferentiated cells.

## Discussion

Increasing evidence indicates that balanced mitochondrial morphology is crucial for embryonic development and cellular differentiation. As part of mitochondrial dynamics, the mitochondrial fission process is mediated by several proteins. Drp1, one of the proteins regulating fission, has been extensively studied *in vivo* and *in vitro*. Homozygous deletion of Drp1 leads to embryonic lethality in mice at day 11.5 dpc, and *Drp1* ^*−/−*^ mice have defects in trophoblast giant cells and cardiomyocytes (Wakabayashi et al., 2009). Furthermore, neural cell-specific (NS) *Drp1* ^*−/−*^ mice die shortly after birth, and primary cultures of NS- *Drp1* ^*−/−*^ mouse forebrain exhibited a decreased number of neurites and defective synapse formation (Ishihara et al., 2009). However, partial reduction of Drp1 in heterozygous knockout mice did not affect mitochondrial and synaptic viability (Manczak et al., 2012). Another study also showed that myogenic differentiation requires NO-dependent repression of Drp1 activity through phosphorylation of Drp1 by G-kinase (De Palma et al., 2010).

Genetic and pharmacological inhibition of Drp1 promotes cardiac mesodermal differentiation of human induced pluripotent stem cells (Hoque et al., 2018). Mitochondrial dynamics also regulates the fate and identity of stem cells by regulating the nuclear transcriptional program (Khacho et al., 2016). In the present study, we provide evidence that stable depletion of Drp1 causes widespread transcriptional changes in SH-SY5Y cells and improves neuronal differentiation *in vitro*. Genes related to neurogenesis, neuronal differentiation, synapse formation, and axon development were upregulated in GO terms. The biological relevance of Drp1 in neuronal differentiation has not been widely studied. Therefore, we analyzed cellular morphology and mitochondrial changes in Drp1 knockdown SH-SY5Y cells during RA-BDNF-induced differentiation. The expression of neuronal markers is a hallmark of RA-BDNF-induced neuronal differentiation. Based on the GO terms analysis results, the expression levels of genes specific for neuronal differentiation increased significantly in differentiated shDrp1 cells compared with those in differentiated control cells. Thus, Drp1 knockdown positively influences the differentiation of SH-SY5Y cells at the transcriptomic level.

Differentiated neuron-like cells are characterized by multiple morphological parameters (Hromadkova et al., 2020; Riegerova et al., 2021). We found that parameters of neuronal differentiation defined by the CSIRO algorithm were significantly increased in differentiated shDrp1 cells compared to undifferentiated shDrp1 cells, and these characteristics were also significantly higher compared to differentiated control cells, suggesting that altered mitochondrial dynamics affect neuronal differentiation. Valenti et al. found similar results after pharmacological inhibition of Drp1 in hippocampal neuronal progenitor cells (NPCs) from mice with Down’s syndrome (DS). DS NPCs exhibit excessive mitochondrial fragmentation and defective neurogenesis. Drp1 inhibition by the neuroprotective mdivi-1 restored mitochondrial network organization and improved neuronal differentiation of DS NPCs, representing an attractive strategy for restoring neurogenesis (Valenti et al., 2017). In hereditary spastic paraplegias (HSPs), the inhibition of Drp1 by a selective peptide inhibitor P110 or genetic depletion of Drp1 rescued axonal and neuronal degeneration in human iPSC derived from the fibroblasts of patients with the SPG11 mutation, implicating mitochondrial dynamics in neurodegeneration (Chen et al., 2022).

Studying the signaling molecules involved in the neuronal differentiation of SH-SY5Y cells, we found similar JNK, Akt, MEK1/2, and p38 activation in both cell lines. At the same time, we observed reduced ERK1/2 phosphorylation in shDrp1 cells after RA-induced differentiation on Day 3, and the total ERK1/2 protein levels were not decreased. MEK1/2, which activates ERK1/2 by phosphorylation, was activated in both cell lines to the same extent, suggesting that the reduced phosphorylation of ERK1/2 may not originate from reduced kinase but elevated phosphatase activity. Dual-specificity phosphatases (DUSPs) control ERK1/2 activity in cells. DUSP1 is known to dephosphorylate ERK1, while DUSP6 dephosphorylates ERK1 and ERK2, rendering them inactive. DUSP6 downregulation results in ERK1/2 activation (Prieto et al., 2016). Inhibition of DUSP1 and DUSP6 by the potent inhibitor BCI resulted in robust phosphorylation of ERK1/2 on Day 3 of differentiation in control but not in shDrp1 cells, confirming the dephosphorylation of ERK1/2 by DUSP1 and 6 in control cells. That suggests that dephosphorylation of ERK1/2 is independent of DUSP1 and 6 in shDrp1 cells.

This alternative scenario may occur through the transcriptional modulation of different DUSP members, leading to the deactivation of ERK1/2. The upregulation of DUSP2 may result in the deactivation of ERK1/2 (Kim et al., 1999; Ding et al., 2022). Our transcriptomic data did not show downregulation of DUSP1 and DUSP6 genes in shDrp1 cells, but DUSP2 gene expression increased significantly in differentiated shDrp1 cells compared to differentiated control cells (Supplementary Figure S6). This proposed mechanism, however, requires further investigation. Nevertheless, our data suggest that the downregulation of Drp1 causes ERK1/2 independent neuronal differentiation in SH-SY5Y cells.

Mitochondrial morphology remodeling and maturation occur during neuronal differentiation to facilitate metabolic reprogramming and adaptation, including several cell types such as embryonic stem cells, induced pluripotent stem cells, and neuroblasts (Voccoli and Colombaioni, 2009; Choi et al., 2015). A recent study also confirmed mitochondrial network remodeling during neuronal differentiation in SH-SY5Y cells, neuroscience’s most commonly used cell model. Mitochondria populations were significantly increased in tubular phenotype during differentiation (D'Aloia et al., 2024). During differentiation, we also monitored mitochondrial network changes and mitochondrial fusion-fission protein levels in our model. During RA-BDNF-induced differentiation, the Opa1 protein gradually but not significantly increased in control. MFN1 protein levels significantly increased, and MFN2 levels did not change during differentiation in control cells. A similar trend occurred in Drp1 knockdown cells; however, MFN1 and MFN2 levels were significantly reduced compared to the respective differentiation stages of control cells. This result indicates that a compensatory mechanism develops in response to Drp1 suppression for the loss/reduction of a crucial fission protein, resulting in altered mitochondrial dynamics.

Posttranslational modifications of Drp1 include reversible phosphorylation. Two extensively studied phosphorylation sites are located at the Ser-616 and Ser-637 of the protein. CDK1-catalyzed phosphorylation of Drp1 at Ser-616 induces microtubule localization of Drp1 and promotes mitochondrial translocation, resulting in mitochondrial division in Jurkat 6E.1 cells (Strack et al., 2013). A panoply of kinases drives the phosphorylation of Drp1 at Ser-637, and the specific role of phosphorylation depends on different parameters, including cell type and upstream regulatory molecules. In most cases, however, the phosphorylation of Ser-637 of Drp1 reduces the catalytic activity of the protein (Jin et al., 2021). In our model, we saw phosphorylated Drp1 at Ser-616 from Day 0 to Day 6. The phosphorylation of Drp1 at Ser-637, which inhibits mitochondrial fission, was induced by RA. This phosphorylation was lost by Day 6. It is known that a distinct Ser616/Ser637 ratio is required to regulate astrocyte mitochondrial fission-fusion (Ko et al., 2016). Furthermore, elevated phosphorylation of Ser-616 alone is not enough to induce mitochondrial fission. In cardiomyocytes, the phosphorylation of Ser-637 regulates Ser-616 phosphorylation (Jhun et al., 2018). It is tempting to speculate that the two sites are required in our control cells for the regulated fusion-fission process during differentiation. Further investigation of Drp1 phosphorylation during differentiation would be fascinating.

After 6 days of differentiation, active mitochondria of control cells were stained with Mitotracker RedCMX Ros, and major rearrangements of mitochondrial networks were observed, resulting from a mix of tubular phenotypes to interconnected tubules. The mitochondrial networks of shDrp1 cells remained highly fused with asymmetric, typically perinuclear compact distribution. We also observed elongated, highly interconnected tubules, suggesting mitochondrial adaptation to neuronal cell morphology.

Increasing mitochondrial bioenergetics parameters based on OCR supports the link between elongated mitochondrial architecture and bioenergetics adaptation in control cells. The reason that we have not seen significant differences in specific parameters might be that we only studied cells after 6 days of RA-BDNF-induced differentiation. D'Aloia et al., 2024 reported that the maximal respiratory capacity was reached on the 20th and maintained to the 40th days of differentiation. They conclude that differentiating cells reached the mitochondrial rearrangement required to mature neurons by day 20. shDrp1 cells exhibited similar behavior to control, but maximal respiration of shDrp1 cells reached a significantly higher OCR by Day 6 compared to control and undifferentiated shDrp1 cells, suggesting that shDrp1 cells acquire a greater mitochondrial respiratory capacity. We speculate that shDrp1 cells might reach a complete mitochondrial rearrangement earlier during differentiation, which might be one reason for improved neuronal cell phenotype.

The downregulation of Drp1 neither inhibited nor enhanced RA, and mitochondrial inhibitors induced cell death. Our results agree with a previously published study demonstrating that Drp1 knockdown delays but does not inhibit apoptosis, and Drp1-dependent mitochondrial fission is not a prerequisite for apoptosis (Estaquier and Arnoult, 2007). Moreover, we demonstrate for the first time that downregulation of Drp1 delays toxic N-Htt aggregate formation suggesting protection of cells from neurotoxicity in an *in vitro* cellular model. We propose that downregulation of Drp1 improves neuronal differentiation via transcriptional modulation of genes responsible for neuronal development and members of the ERK1/2 signaling pathway and by rearranging mitochondrial dynamics in undifferentiated cell states.

## Conclusion

The current study investigated the role of Drp1, a major mitochondrial fission protein, in neuronal differentiation of SH-SY5Y cells. Stable knockdown of Drp1 resulted in mitochondrial network rearrangement and the upregulation of genes involved in neural development, including synapse assembly, neurogenesis, differentiation, and morphogenesis. Knockdown of Drp1 improved neuronal differentiation, resulting in longer neurite outgrowth, higher segmentation, and a higher number of extremities. RA-BDNF-induced neuronal differentiation was associated with a significant reduction of ERK1/2 phosphorylation suggesting an ERK1/2-independent neuronal differentiation of shDrp1 cells. Furthermore, the phosphorylation status of ERK1/2 was independent of the dual specificity phosphatases DUSP1/6 in shDrp1 cells. Highly fused and elongated mitochondrial structures were maintained during differentiation in shDrp1 cells, and we observed significantly elevated maximal mitochondrial respiration. These results demonstrate that the downregulation of Drp1 improves neuronal differentiation through transcriptional changes and mitochondrial rearrangement of undifferentiated cells. The shDrp1 cells responded to apoptotic stimuli similar to control *in vitro*. Notably, the formation of toxic aggregates of the mutant huntingtin (mHtt) protein was reduced after overexpression of the N-terminal mHtt in shDrp1 cells. Thus, reducing Drp1 in a regulated manner may improve cell survival and prevent neurotoxicity. This mechanism should be investigated further to identify novel strategies to combat neurodegenerative diseases.

## Data Availability

RNA seq data is available under the BioProject accession number: PRJNA1071584 on the following links: https://www.ncbi.nlm.nih.gov/sra/PRJNA1071584 https://dataview.ncbi.nlm.nih.gov/object/PRJNA1071584?reviewer=44maot4vpk953ovqqigtvdavjp. Data supporting reported results can be found at the Department of Medical Chemistry http://193.6.152.202:5000/ accessed on 6 March 2024. Requests to access the datasets should be directed to the corresponding author.

## References

[B1] Agostini M. Romeo F. Inoue S. Niklison-Chirou M. V. Elia A. J. Dinsdale D. (2016). Metabolic reprogramming during neuronal differentiation. Cell Death Differ. 23 (9), 1502–1514. 10.1038/cdd.2016.36 27058317 PMC5072427

[B2] Aladdin A. Yao Y. Yang C. Kahlert G. Ghani M. Kiraly N. (2020). The proteasome activators blm10/pa200 enhance the proteasomal degradation of N-terminal huntingtin. Biomolecules 10 (11), 1581. 10.3390/biom10111581 33233776 PMC7699873

[B3] Al Ojaimi M. Salah A. El-Hattab A. W. (2022). Mitochondrial fission and fusion: molecular mechanisms, biological functions, and related disorders. Membr. (Basel) 12 (9), 893. 10.3390/membranes12090893 PMC9502208 36135912

[B4] Attoff K. Johansson Y. Cediel-Ulloa A. Lundqvist J. Gupta R. Caiment F. (2020). Acrylamide alters CREB and retinoic acid signalling pathways during differentiation of the human neuroblastoma SH-SY5Y cell line. Sci. Rep. 10 (1), 16714. 10.1038/s41598-020-73698-6 33028897 PMC7541504

[B5] Beal M. F. (2005). Mitochondria take center stage in aging and neurodegeneration. Ann. Neurol. 58 (4), 495–505. 10.1002/ana.20624 16178023

[B6] Beckervordersandforth R. Ebert B. Schaffner I. Moss J. Fiebig C. Shin J. (2017). Role of mitochondrial metabolism in the control of early lineage progression and aging phenotypes in adult hippocampal neurogenesis. Neuron 93 (3), 560–573. 10.1016/j.neuron.2016.12.017 28111078 PMC5300896

[B7] Bleazard W. McCaffery J. M. King E. J. Bale S. Mozdy A. Tieu Q. (1999). The dynamin-related GTPase Dnm1 regulates mitochondrial fission in yeast. Nat. Cell Biol. 1 (5), 298–304. 10.1038/13014 10559943 PMC3739991

[B8] Chen Z. Chai E. Mou Y. Roda R. H. Blackstone C. Li X. J. (2022). Inhibiting mitochondrial fission rescues degeneration in hereditary spastic paraplegia neurons. Brain 145 (11), 4016–4031. 10.1093/brain/awab488 35026838 PMC10200290

[B9] Cheng B. Martinez A. A. Morado J. Scofield V. Roberts J. L. Maffi S. K. (2013). Retinoic acid protects against proteasome inhibition associated cell death in SH-SY5Y cells via the AKT pathway. Neurochem. Int. 62 (1), 31–42. 10.1016/j.neuint.2012.10.014 23142153

[B10] Cheung Y. T. Lau W. K. Yu M. S. Lai C. S. Yeung S. C. So K. F. (2009). Effects of all-trans-retinoic acid on human SH-SY5Y neuroblastoma as *in vitro* model in neurotoxicity research. Neurotoxicology 30 (1), 127–135. 10.1016/j.neuro.2008.11.001 19056420

[B11] Choi H. W. Kim J. H. Chung M. K. Hong Y. J. Jang H. S. Seo B. J. (2015). Mitochondrial and metabolic remodeling during reprogramming and differentiation of the reprogrammed cells. Stem Cells Dev. 24 (11), 1366–1373. 10.1089/scd.2014.0561 25590788

[B12] Costa V. Giacomello M. Hudec R. Lopreiato R. Ermak G. Lim D. (2010). Mitochondrial fission and cristae disruption increase the response of cell models of Huntington's disease to apoptotic stimuli. EMBO Mol. Med. 2 (12), 490–503. 10.1002/emmm.201000102 21069748 PMC3044888

[B13] D'Aloia A. Pastori V. Blasa S. Campioni G. Peri F. Sacco E. (2024). A new advanced cellular model of functional cholinergic-like neurons developed by reprogramming the human SH-SY5Y neuroblastoma cell line. Cell Death Discov. 10 (1), 24. 10.1038/s41420-023-01790-7 38216593 PMC10786877

[B14] de Medeiros L. M. De Bastiani M. A. Rico E. P. Schonhofen P. Pfaffenseller B. Wollenhaupt-Aguiar B. (2019). Cholinergic differentiation of human neuroblastoma SH-SY5Y cell line and its potential use as an *in vitro* model for alzheimer's disease studies. Mol. Neurobiol. 56 (11), 7355–7367. 10.1007/s12035-019-1605-3 31037648

[B15] De Palma C. Falcone S. Pisoni S. Cipolat S. Panzeri C. Pambianco S. (2010). Nitric oxide inhibition of Drp1-mediated mitochondrial fission is critical for myogenic differentiation. Cell Death Differ. 17 (11), 1684–1696. 10.1038/cdd.2010.48 20467441 PMC3050583

[B16] Ding S. Gao Y. Lv D. Tao Y. Liu S. Chen C. (2022). DNTTIP1 promotes nasopharyngeal carcinoma metastasis via recruiting HDAC1 to DUSP2 promoter and activating ERK signaling pathway. EBioMedicine 81, 104100. 10.1016/j.ebiom.2022.104100 35689852 PMC9189780

[B17] Douida A. Batista F. Boto P. Regdon Z. Robaszkiewicz A. Tar K. (2021). Cells lacking PA200 adapt to mitochondrial dysfunction by enhancing glycolysis via distinct Opa1 processing. Int. J. Mol. Sci. 22 (4), 1629. 10.3390/ijms22041629 33562813 PMC7914502

[B18] Douida A. Batista F. Robaszkiewicz A. Boto P. Aladdin A. Szenykiv M. (2020). The proteasome activator PA200 regulates expression of genes involved in cell survival upon selective mitochondrial inhibition in neuroblastoma cells. J. Cell Mol. Med. 24 (12), 6716–6730. 10.1111/jcmm.15323 32368861 PMC7299700

[B19] Dutta S. Pal D. Rao M. R. S. (2023). Retinoic acid-mediated differentiation of mouse embryonic stem cells to neuronal cells. Methods Mol. Biol. 2736, 39–51. 10.1007/7651_2023_480 37140812

[B20] Estaquier J. Arnoult D. (2007). Inhibiting Drp1-mediated mitochondrial fission selectively prevents the release of cytochrome c during apoptosis. Cell Death Differ. 14 (6), 1086–1094. 10.1038/sj.cdd.4402107 17332775

[B21] Forster J. I. Köglsberger S. Trefois C. Boyd O. Baumuratov A. S. Buck L. (2016). Characterization of differentiated SH-SY5Y as neuronal screening model reveals increased oxidative vulnerability. J. Biomol. Screen 21 (5), 496–509. 10.1177/1087057115625190 26738520 PMC4904349

[B22] Garza-Lopez E. Vue Z. Katti P. Neikirk K. Biete M. Lam J. (2021). Protocols for generating surfaces and measuring 3D organelle morphology using Amira. Cells 11 (1), 65. 10.3390/cells11010065 35011629 PMC8750564

[B23] Guo X. Disatnik M. H. Monbureau M. Shamloo M. Mochly-Rosen D. Qi X. (2013). Inhibition of mitochondrial fragmentation diminishes Huntington's disease-associated neurodegeneration. J. Clin. Invest. 123 (12), 5371–5388. 10.1172/jci70911 24231356 PMC3859413

[B24] Haun F. Nakamura T. Shiu A. D. Cho D. H. Tsunemi T. Holland E. A. (2013). S-nitrosylation of dynamin-related protein 1 mediates mutant huntingtin-induced mitochondrial fragmentation and neuronal injury in Huntington's disease. Antioxid. Redox Signal 19 (11), 1173–1184. 10.1089/ars.2012.4928 23641925 PMC3785802

[B25] He Y. Kastin A. J. Hsuchou H. Pan W. (2009). The Cdk5/p35 kinases modulate leptin-induced STAT3 signaling. J. Mol. Neurosci. 39 (1-2), 49–58. 10.1007/s12031-008-9174-3 19156541 PMC2745521

[B26] Hoque A. Sivakumaran P. Bond S. T. Ling N. X. Y. Kong A. M. Scott J. W. (2018). Mitochondrial fission protein Drp1 inhibition promotes cardiac mesodermal differentiation of human pluripotent stem cells. Cell Death Discov. 4, 39. 10.1038/s41420-018-0042-9 PMC5841367 29531836

[B27] Hromadkova L. Bezdekova D. Pala J. Schedin-Weiss S. Tjernberg L. O. Hoschl C. (2020). Brain-derived neurotrophic factor (BDNF) promotes molecular polarization and differentiation of immature neuroblastoma cells into definitive neurons. Biochim. Biophys. Acta Mol. Cell Res. 1867 (9), 118737. 10.1016/j.bbamcr.2020.118737 32389647

[B28] Ioghen O. C. Ceafalan L. C. Popescu B. O. (2023). SH-SY5Y cell line *in vitro* models for Parkinson disease research-old practice for new trends. J. Integr. Neurosci. 22 (1), 20. 10.31083/j.jin2201020 36722247

[B29] Ishihara N. Nomura M. Jofuku A. Kato H. Suzuki S. O. Masuda K. (2009). Mitochondrial fission factor Drp1 is essential for embryonic development and synapse formation in mice. Nat. Cell Biol. 11 (8), 958–966. 10.1038/ncb1907 19578372

[B30] Jhun B. S. Adaniya S. M. Mancini T. J. Cao J. L. King M. E. Sheu S. S. (2018). Protein kinase D activation induces mitochondrial fragmentation and dysfunction in cardiomyocytes. J. Physiol. 596 (5), 827–855. 10.1113/JP275418 29313986 PMC5830422

[B31] Jin J. Y. Wei X. X. Zhi X. L. Wang X. H. Meng D. (2021). Drp1-dependent mitochondrial fission in cardiovascular disease. Acta Pharmacol. Sin. 42 (5), 655–664. 10.1038/s41401-020-00518-y 32913266 PMC8115655

[B32] Kang J. B. Koh P. O. (2023). Retinoic acid has neuroprotective effects by modulating thioredoxin in ischemic brain damage and glutamate-exposed neurons. Neuroscience 521, 166–181. 10.1016/j.neuroscience.2023.04.028 37149281

[B33] Kaplan D. R. Matsumoto K. Lucarelli E. Thiele C. J. (1993). Induction of TrkB by retinoic acid mediates biologic responsiveness to BDNF and differentiation of human neuroblastoma cells. Eukaryotic Signal Transduction Group. Neuron 11 (2), 321–331. 10.1016/0896-6273(93)90187-v 8394722

[B34] Khacho M. Clark A. Svoboda D. S. Azzi J. MacLaurin J. G. Meghaizel C. (2016). Mitochondrial dynamics impacts stem cell identity and fate decisions by regulating a nuclear transcriptional program. Cell Stem Cell 19 (2), 232–247. 10.1016/j.stem.2016.04.015 27237737

[B35] Khacho M. Slack R. S. (2018). Mitochondrial dynamics in the regulation of neurogenesis: from development to the adult brain. Dev. Dyn. 247 (1), 47–53. 10.1002/dvdy.24538 28643345

[B36] Kim S. C. Hahn J. S. Min Y. H. Yoo N. C. Ko Y. W. Lee W. J. (1999). Constitutive activation of extracellular signal-regulated kinase in human acute leukemias: combined role of activation of MEK, hyperexpression of extracellular signal-regulated kinase, and downregulation of a phosphatase, PAC1. Blood 93 (11), 3893–3899. 10.1182/blood.v93.11.3893.407k14_3893_3899 10339498

[B37] Kitamura S. Yanagi T. Imafuku K. Hata H. Abe R. Shimizu H. (2017). Drp1 regulates mitochondrial morphology and cell proliferation in cutaneous squamous cell carcinoma. J. Dermatol Sci. 88 (3), 298–307. 10.1016/j.jdermsci.2017.08.004 28818497

[B38] Ko A. R. Hyun H. W. Min S. J. Kim J. E. (2016). The differential DRP1 phosphorylation and mitochondrial dynamics in the regional specific astroglial death induced by status epilepticus. Front. Cell Neurosci. 10, 124. 10.3389/fncel.2016.00124 27242436 PMC4870264

[B39] Koirala S. Guo Q. Kalia R. Bui H. T. Eckert D. M. Frost A. (2013). Interchangeable adaptors regulate mitochondrial dynamin assembly for membrane scission. Proc. Natl. Acad. Sci. U. S. A. 110 (15), E1342–E1351. 10.1073/pnas.1300855110 23530241 PMC3625255

[B40] Korecka J. A. van Kesteren R. E. Blaas E. Spitzer S. O. Kamstra J. H. Smit A. B. (2013). Phenotypic characterization of retinoic acid differentiated SH-SY5Y cells by transcriptional profiling. PLoS One 8 (5), e63862. 10.1371/journal.pone.0063862 23724009 PMC3665836

[B41] Kovalevich J. Langford D. (2013). Considerations for the use of SH-SY5Y neuroblastoma cells in neurobiology. Methods Mol. Biol. 1078, 9–21. 10.1007/978-1-62703-640-5_2 23975817 PMC5127451

[B42] Lee J. H. Kim K. T. (2004). Induction of cyclin-dependent kinase 5 and its activator p35 through the extracellular-signal-regulated kinase and protein kinase A pathways during retinoic-acid mediated neuronal differentiation in human neuroblastoma SK-N-BE(2)C cells. J. Neurochem. 91 (3), 634–647. 10.1111/j.1471-4159.2004.02770.x 15485494

[B43] Livak K. J. Schmittgen T. D. (2001). Analysis of relative gene expression data using real-time quantitative PCR and the 2(-Delta Delta C(T)) Method. Methods 25 (4), 402–408. 10.1006/meth.2001.1262 11846609

[B44] Lopez-Carballo G. Moreno L. Masia S. Perez P. Barettino D. (2002). Activation of the phosphatidylinositol 3-kinase/Akt signaling pathway by retinoic acid is required for neural differentiation of SH-SY5Y human neuroblastoma cells. J. Biol. Chem. 277 (28), 25297–25304. 10.1074/jbc.M201869200 12000752

[B45] Manczak M. Sesaki H. Kageyama Y. Reddy P. H. (2012). Dynamin-related protein 1 heterozygote knockout mice do not have synaptic and mitochondrial deficiencies. Biochimica biophysica acta 1822 (6), 862–874. 10.1016/j.bbadis.2012.02.017 PMC3338881 22387883

[B46] Mao K. Wang K. Liu X. Klionsky D. J. (2013). The scaffold protein Atg11 recruits fission machinery to drive selective mitochondria degradation by autophagy. Dev. Cell 26 (1), 9–18. 10.1016/j.devcel.2013.05.024 23810512 PMC3720741

[B47] Miloso M. Villa D. Crimi M. Galbiati S. Donzelli E. Nicolini G. (2004). Retinoic acid-induced neuritogenesis of human neuroblastoma SH-SY5Y cells is ERK independent and PKC dependent. J. Neurosci. Res. 75 (2), 241–252. 10.1002/jnr.10848 14705145

[B48] Mishra P. Chan D. C. (2016). Metabolic regulation of mitochondrial dynamics. J. Cell Biol. 212 (4), 379–387. 10.1083/jcb.201511036 26858267 PMC4754720

[B49] Narain Y. Wyttenbach A. Rankin J. Furlong R. A. Rubinsztein D. C. (1999). A molecular investigation of true dominance in Huntington's disease. J. Med. Genet. 36 (10), 739–746. 10.1136/jmg.36.10.739 10528852 PMC1734229

[B50] Noy N. (2010). Between death and survival: retinoic acid in regulation of apoptosis. Annu. Rev. Nutr. 30, 201–217. 10.1146/annurev.nutr.28.061807.155509 20415582

[B51] Nunnari J. Suomalainen A. (2012). Mitochondria: in sickness and in health. Cell 148 (6), 1145–1159. 10.1016/j.cell.2012.02.035 22424226 PMC5381524

[B52] Okamoto K. Shaw J. M. (2005). Mitochondrial morphology and dynamics in yeast and multicellular eukaryotes. Annu. Rev. Genet. 39, 503–536. 10.1146/annurev.genet.38.072902.093019 16285870

[B53] Otera H. Ishihara N. Mihara K. (2013). New insights into the function and regulation of mitochondrial fission. Biochimica biophysica acta 1833 (5), 1256–1268. 10.1016/j.bbamcr.2013.02.002 23434681

[B54] Park J. C. Jeong W. J. Kim M. Y. Min D. Choi K. Y. (2016). Retinoic-acid-mediated HRas stabilization induces neuronal differentiation of neural stem cells during brain development. J. Cell Sci. 129 (15), 2997–3007. 10.1242/jcs.184366 27185863

[B55] Prieto J. Leon M. Ponsoda X. Sendra R. Bort R. Ferrer-Lorente R. (2016). Early ERK1/2 activation promotes DRP1-dependent mitochondrial fission necessary for cell reprogramming. Nat. Commun. 7, 11124. 10.1038/ncomms11124 27030341 PMC4821885

[B56] Qi Z. Huang Z. Xie F. Chen L. (2019). Dynamin-related protein 1: a critical protein in the pathogenesis of neural system dysfunctions and neurodegenerative diseases. J. Cell Physiol. 234 (7), 10032–10046. 10.1002/jcp.27866 30515821

[B57] Reddy P. H. (2008). Mitochondrial medicine for aging and neurodegenerative diseases. Neuromolecular Med. 10 (4), 291–315. 10.1007/s12017-008-8044-z 18566920 PMC3235551

[B58] Riegerova P. Brejcha J. Bezdekova D. Chum T. Masinova E. Cermakova N. (2021). Expression and localization of AβPP in SH-SY5Y cells depends on differentiation state. J. Alzheimers Dis. 82 (2), 485–491. 10.3233/JAD-201409 34057078 PMC8385523

[B59] Ross R. A. Spengler B. A. Biedler J. L. (1983). Coordinate morphological and biochemical interconversion of human neuroblastoma cells. J. Natl. Cancer Inst. 71 (4), 741–747. 6137586

[B60] Samangouei P. Crespo-Avilan G. E. Cabrera-Fuentes H. Hernández-Reséndiz S. Ismail N. I. Katwadi K. B. (2018). MiD49 and MiD51: new mediators of mitochondrial fission and novel targets for cardioprotection. Cond. Med. 1 (5), 239–246. 30338314 PMC6191188

[B61] Schiro G. Iacono S. Ragonese P. Aridon P. Salemi G. Balistreri C. R. (2022). A brief overview on BDNF-trk pathway in the nervous system: a potential biomarker or possible target in treatment of multiple sclerosis? Front. Neurol. 13, 917527. 10.3389/fneur.2022.917527 35911894 PMC9332890

[B62] Scott I. Youle R. J. (2010). Mitochondrial fission and fusion. Essays Biochem. 47, 85–98. 10.1042/bse0470085 20533902 PMC4762097

[B63] Sesaki H. Jensen R. E. (1999). Division versus fusion: dnm1p and Fzo1p antagonistically regulate mitochondrial shape. J. Cell Biol. 147 (4), 699–706. 10.1083/jcb.147.4.699 10562274 PMC2156171

[B64] Shipley M. M. Mangold C. A. Szpara M. L. (2016). Differentiation of the SH-SY5Y human neuroblastoma cell line. J. Vis. Exp. 108, 53193. 10.3791/53193 PMC4828168 26967710

[B65] Shirendeb U. P. Calkins M. J. Manczak M. Anekonda V. Dufour B. McBride J. L. (2012). Mutant huntingtin's interaction with mitochondrial protein Drp1 impairs mitochondrial biogenesis and causes defective axonal transport and synaptic degeneration in Huntington's disease. Hum. Mol. Genet. 21 (2), 406–420. 10.1093/hmg/ddr475 21997870 PMC3276281

[B66] Singh U. S. Pan J. Kao Y. L. Joshi S. Young K. L. Baker K. M. (2003). Tissue transglutaminase mediates activation of RhoA and MAP kinase pathways during retinoic acid-induced neuronal differentiation of SH-SY5Y cells. J. Biol. Chem. 278 (1), 391–399. 10.1074/jbc.M206361200 12401808

[B67] Song W. Chen J. Petrilli A. Liot G. Klinglmayr E. Zhou Y. (2011). Mutant huntingtin binds the mitochondrial fission GTPase dynamin-related protein-1 and increases its enzymatic activity. Nat. Med. 17 (3), 377–382. 10.1038/nm.2313 21336284 PMC3051025

[B68] Strack S. Wilson T. J. Cribbs J. T. (2013). Cyclin-dependent kinases regulate splice-specific targeting of dynamin-related protein 1 to microtubules. J. Cell Biol. 201 (7), 1037–1051. 10.1083/jcb.201210045 23798729 PMC3691453

[B69] Su B. Wang X. Zheng L. Perry G. Smith M. A. Zhu X. (2010). Abnormal mitochondrial dynamics and neurodegenerative diseases. Biochimica biophysica acta 1802 (1), 135–142. 10.1016/j.bbadis.2009.09.013 PMC2790543 19799998

[B70] Suen D. F. Norris K. L. Youle R. J. (2008). Mitochondrial dynamics and apoptosis. Genes Dev. 22 (12), 1577–1590. 10.1101/gad.1658508 18559474 PMC2732420

[B71] Sutovsky P. Navara C. S. Schatten G. (1996). Fate of the sperm mitochondria, and the incorporation, conversion, and disassembly of the sperm tail structures during bovine fertilization. Biol. reproduction 55 (6), 1195–1205. 10.1095/biolreprod55.6.1195 8949874

[B72] Taguchi N. Ishihara N. Jofuku A. Oka T. Mihara K. (2007). Mitotic phosphorylation of dynamin-related GTPase Drp1 participates in mitochondrial fission. J. Biol. Chem. 282 (15), 11521–11529. 10.1074/jbc.M607279200 17301055

[B73] Tanwar D. K. Parker D. J. Gupta P. Spurlock B. Alvarez R. D. Basu M. K. (2016). Crosstalk between the mitochondrial fission protein, Drp1, and the cell cycle is identified across various cancer types and can impact survival of epithelial ovarian cancer patients. Oncotarget 7 (37), 60021–60037. 10.18632/oncotarget.11047 27509055 PMC5312366

[B74] Teppola H. Sarkanen J. R. Jalonen T. O. Linne M. L. (2016). Morphological differentiation towards neuronal phenotype of SH-SY5Y neuroblastoma cells by estradiol, retinoic acid and cholesterol. Neurochem. Res. 41 (4), 731–747. 10.1007/s11064-015-1743-6 26518675 PMC4824837

[B75] Tieu Q. Okreglak V. Naylor K. Nunnari J. (2002). The WD repeat protein, Mdv1p, functions as a molecular adaptor by interacting with Dnm1p and Fis1p during mitochondrial fission. J. Cell Biol. 158 (3), 445–452. 10.1083/jcb.200205031 12163467 PMC2173813

[B76] Torocsik D. Fazekas F. Poliska S. Gregus A. Janka E. A. Dull K. (2021). Epidermal growth factor modulates palmitic acid-induced inflammatory and lipid signaling pathways in SZ95 sebocytes. Front. Immunol. 12, 600017. 10.3389/fimmu.2021.600017 34025636 PMC8134683

[B77] Truckenmiller M. E. Vawter M. P. Cheadle C. Coggiano M. Donovan D. M. Freed W. J. (2001). Gene expression profile in early stage of retinoic acid-induced differentiation of human SH-SY5Y neuroblastoma cells. Restor. Neurol. Neurosci. 18 (2-3), 67–80. 11847429

[B78] Twig G. Shirihai O. S. (2011). The interplay between mitochondrial dynamics and mitophagy. Antioxid. Redox Signal 14 (10), 1939–1951. 10.1089/ars.2010.3779 21128700 PMC3078508

[B79] Valenti D. Rossi L. Marzulli D. Bellomo F. De Rasmo D. Signorile A. (2017). Inhibition of Drp1-mediated mitochondrial fission improves mitochondrial dynamics and bioenergetics stimulating neurogenesis in hippocampal progenitor cells from a Down syndrome mouse model. Biochim. Biophys. Acta Mol. Basis Dis. 1863 (12), 3117–3127. 10.1016/j.bbadis.2017.09.014 28939434

[B80] Vantaggiato C. Castelli M. Giovarelli M. Orso G. Bassi M. T. Clementi E. (2019). The fine tuning of drp1-dependent mitochondrial remodeling and autophagy controls neuronal differentiation. Front. Cell Neurosci. 13, 120. 10.3389/fncel.2019.00120 31019453 PMC6458285

[B81] Vichai V. Kirtikara K. (2006). Sulforhodamine B colorimetric assay for cytotoxicity screening. Nat. Protoc. 1 (3), 1112–1116. 10.1038/nprot.2006.179 17406391

[B82] Voccoli V. Colombaioni L. (2009). Mitochondrial remodeling in differentiating neuroblasts. Brain Res. 1252, 15–29. 10.1016/j.brainres.2008.11.026 19071097

[B83] Wai T. Langer T. (2016). Mitochondrial dynamics and metabolic regulation. Trends Endocrinol. Metab. 27 (2), 105–117. 10.1016/j.tem.2015.12.001 26754340

[B84] Wakabayashi J. Zhang Z. Wakabayashi N. Tamura Y. Fukaya M. Kensler T. W. (2009). The dynamin-related GTPase Drp1 is required for embryonic and brain development in mice. J. Cell Biol. 186 (6), 805–816. 10.1083/jcb.200903065 19752021 PMC2753156

[B85] Wang D. Lagerstrom R. Sun C. Bishof L. Valotton P. Gotte M. (2010). HCA-vision: automated neurite outgrowth analysis. J. Biomol. Screen 15 (9), 1165–1170. 10.1177/1087057110382894 20855562

[B86] Wang H. Lim P. J. Karbowski M. Monteiro M. J. (2009). Effects of overexpression of huntingtin proteins on mitochondrial integrity. Hum. Mol. Genet. 18 (4), 737–752. 10.1093/hmg/ddn404 19039036 PMC2722218

[B87] Wolf G. (2008). Retinoic acid as cause of cell proliferation or cell growth inhibition depending on activation of one of two different nuclear receptors. Nutr. Rev. 66 (1), 55–59. 10.1111/j.1753-4887.2007.00006.x 18254885

[B88] Yaworsky P. J. Kappen C. (1999). Heterogeneity of neural progenitor cells revealed by enhancers in the nestin gene. Dev. Biol. 205 (2), 309–321. 10.1006/dbio.1998.9035 9917366 PMC3938161

[B89] Youle R. J. van der Bliek A. M. (2012). Mitochondrial fission, fusion, and stress. Science 337 (6098), 1062–1065. 10.1126/science.1219855 22936770 PMC4762028

[B90] Yu Y. M. Han P. L. Lee J. K. (2003). JNK pathway is required for retinoic acid-induced neurite outgrowth of human neuroblastoma, SH-SY5Y. Neuroreport 14 (7), 941–945. 10.1097/01.wnr.0000074341.81633.b8 12802179

[B91] Zheng X. Boyer L. Jin M. Mertens J. Kim Y. Ma L. (2016). Metabolic reprogramming during neuronal differentiation from aerobic glycolysis to neuronal oxidative phosphorylation. Elife 5, e13374. 10.7554/eLife.13374 27282387 PMC4963198

