# Supplementary material for: Stable knockdown of Drp1 improves retinoic acid-BDNF-induced neuronal differentiation through global transcriptomic changes and results in reduced phosphorylation of ERK1/2 independently of DUSP1 and 6
Source: Front Cell Dev Biol. 2024 Mar 14;12:1342741. doi: 10.3389/fcell.2024.1342741 (PMC10972930; doi:10.3389/fcell.2024.1342741)
Supplement: Supplementary file 4 [file DataSheet1.docx]

Supplementary Material

**Stable Knockdown of Drp1 Improves Retinoic Acid-BDNF-Induced Neuronal Differentiation through Global Transcriptomic Changes and Results in Reduced Phosphorylation of ERK1/2 Independently of DUSP1 and 6**

**Marvi Ghani^1,2^, Peleg Zohar^1^, Gyula Újlaki^1,2^, Melinda Tóth^1^, Hailemariam Amsalu^1,2^, Szilárd Póliska^3^, Krisztina Tar^1^ ***

*** Correspondence:** Krisztina Tar, [tark@med.unideb.hu](mailto:tark@med.unideb.hu)

**Table S1. The shRNA clones used in this study**

| Gene ID | Gene symbol | Species | Vector | Marker | Sense strand sequence | Start | End | Target |
| --- | --- | --- | --- | --- | --- | --- | --- | --- |
| 10059 | DNM1L | Human | pGIPZ | GFP | GTAAATTTCTTCACACCAA | 436 | 454 | CDS |
| none | GIPZ empty vector |  | pGIPZ | GFP | Empty pGIPZ vector  (no shRNA) | - | - | - |

**Table S2. List of defined parameters for neurite outgrowth analysis**

| Terms | Definitions |
| --- | --- |
| Maximum neurite length (µm) | The length of the longest path from a neuron body to an extreme segment. |
| Total neurite length (µm) | The sum of the length of each neurite segment for the cell. |
| Number of roots | The number of points where neurite structures touch a neuron body. |
| Number of nodes type 1 | The number of branching points type 1 of each neurite tree. |
| Number of nodes type 2 | The number of branching points type 2 of each neurite tree. |
| Number of segments | A linear structure between branching points or a neuron body. |
| Neurite segment number per cell | Selected neurite segments/number of objects. |
| Segment length vs. total neurite length | Selected neurite segments/total neurite length. |
| Selected neurite segments – branch level | All neurite segments present in a neurite tree. |
| Number of extremities | The number of terminating neurite segments. |
| Number of extremities vs. cell number | The number of extremities/number of objects |

**Table S3. List of primers used in this study**

| Gene Name | Forward Sequence | Reverse Sequence | Ref. |
| --- | --- | --- | --- |
| hGAPDH | GAGTCAACGGATTTGGTCGT | GATCTCGCTCCTGGAAGATG | (Forster, Köglsberger et al. 2016) |
| hDDC | GCAAGTGAATTCCGAAGGAG | CCCCAGGCATGATTATCTTC | (Forster, Köglsberger et al. 2016) |
| hACHE | CCTCCTTGGACGTGTACGAT | AAACAGCGTCACTGATGTCG | (Forster, Köglsberger et al. 2016) |
| hNES | AACAGCGACGGAGGTCTCTA | TTCTCTTGTCCCGCAGACTT | (Forster, Köglsberger et al. 2016) |
| hPAX6 | TGTCCAACGGATGTGTGAGT | TTTCCCAAGCAAAGATGGAC | (Forster, Köglsberger et al. 2016) |
| hSNAP25 | CTGCTCGTGTAGTGGACGAA | CGATCTGGCGATTCTGTGTA | (Forster, Köglsberger et al. 2016) |
| hSOX2 | AACCCCAAGATGCACAACTC | CGGGGCCGGTATTTATAATC | (Forster, Köglsberger et al. 2016) |
| hSYN1 | AATACTGGCTCTGCGATGCT | TGACCACGAGCTCTACGATG | (Forster, Köglsberger et al. 2016) |
| hTUBB3 | CATCCAGAGCAAGAACAGCA | CTCGGTGAACTCCATCTCGT | (Forster, Köglsberger et al. 2016) |
| hPPIA | TTCATCTGCACTGCCAAGAC | TCGAGTTGTCCACAGTCAGC | (Forster, Köglsberger et al. 2016) |
| hβ-Actin | GACCCAGATCATGTTTGAGACC | CATCACGATGCCAGTGGTAC | (Nagy, Docsa et al. 2013) |
| hDNM1L  Primer #1 | CGCTGTCACTGCTGCTAATA | GACCATCTGGATCTACCTCTCT | This study |
| hDNM1L  Primer #2 | GGTCCTCGTCCTGCTTTATTT | GGGCTCTTCTAGACGTTTGATT | This study |

**Table S4. List of antibodies used in this study**

| Antibody | Source | Catalog Number | Dilution |
| --- | --- | --- | --- |
| Drp1 | BD Biosciences | 611112 | 1:1000 |
| MFN1 | Abnova | HOOO55669 M04 | 1:1000 |
| MFN2 | Sigma Aldrich | WH0009927M3 | 1:1000 |
| Opa1 | Novus Biologicals | NB110-55290 | 1:1000 |
| Akt | Cell Signaling | 4691S | 1:1000 |
| P-Akt | Cell Signaling | 4060S | 1:500 |
| SAPK/JNK | Cell Signaling | 9252S | 1:1000 |
| P-SAPK/JNK | Cell Signaling | 9251S | 1:500 |
| P38 MAPK | Cell Signaling | 9212S | 1:1000 |
| P- P38 MAPK | Cell Signaling | 9211S | 1:500 |
| ERK1/2 | Cell Signaling | 9102S | 1:1000 |
| P-ERK1/2 | Cell Signaling | 9101S | 1:500 |
| MEK | Cell Signaling | 9122 | 1:1000 |
| P-MEK | Cell Signaling | 9121 | 1:500 |
| Tuj1 | Biolegend | 801202 | 1:1000 |
| Anti-HA (c29F4) | Cell signaling | 3724s | 1:1000 |
| Phospho-Drp1 S616 | Cell signaling | 3455 | 1:500 |
| Phospho-Drp1 S637 | Cell signaling | 6319 | 1:500 |

**
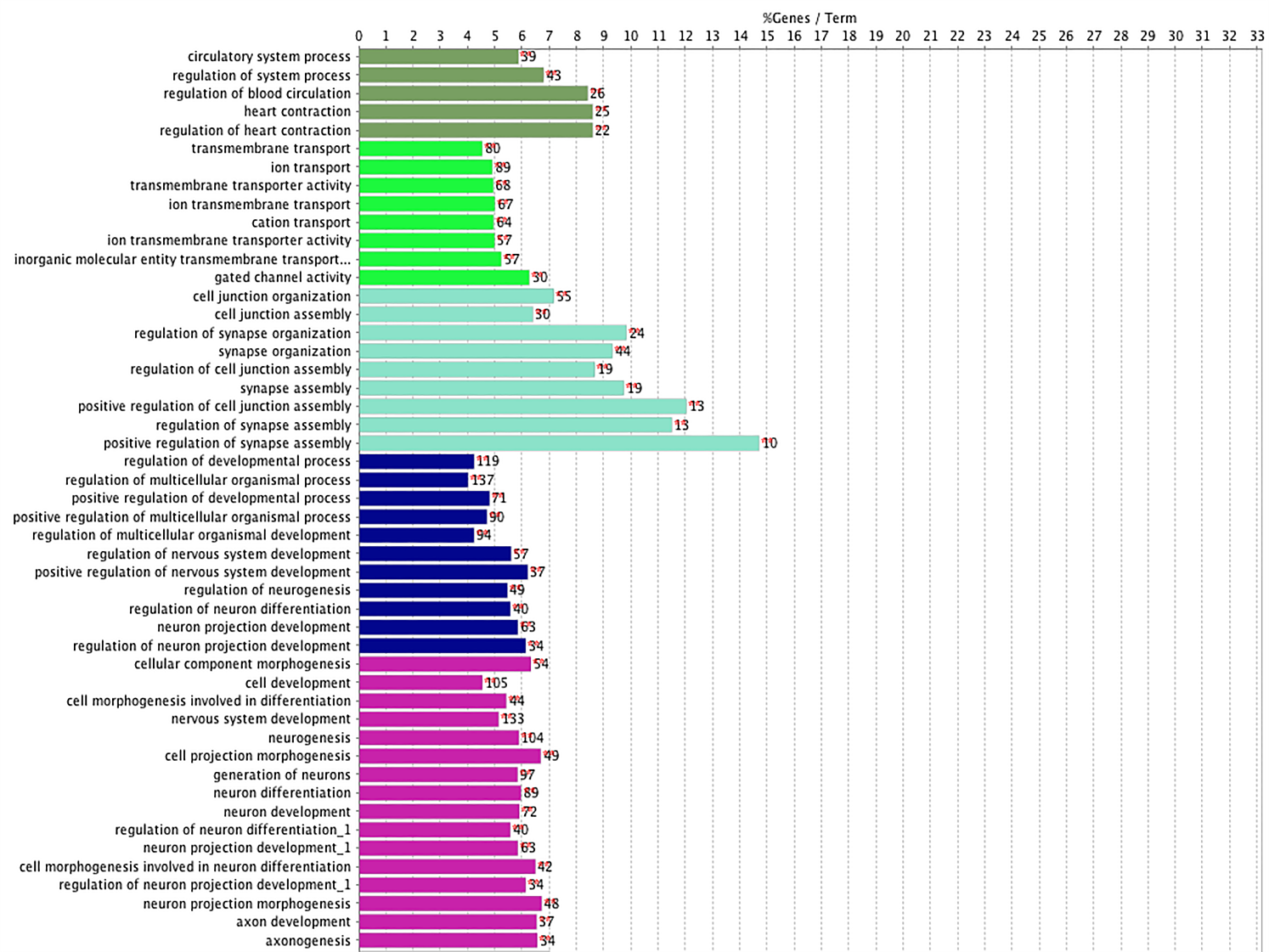
**

**Figure S1. Separate GO analysis of upregulated genes in shDrp1 cells compared to control cells.** Overrepresented GO terms (biological processes) of upregulated genes in shDrp1 cells are shown. Bar graphs represent the associated genes' numbers for each significant GO term and %Genes/Term for upregulated genes. CytoScape 3.4.0 with the ClueGo application was used for gene ontology analysis of differentially expressed genes. A hypergeometric test with Bonferroni step-down correction was used to determine overrepresented GO categories.

**
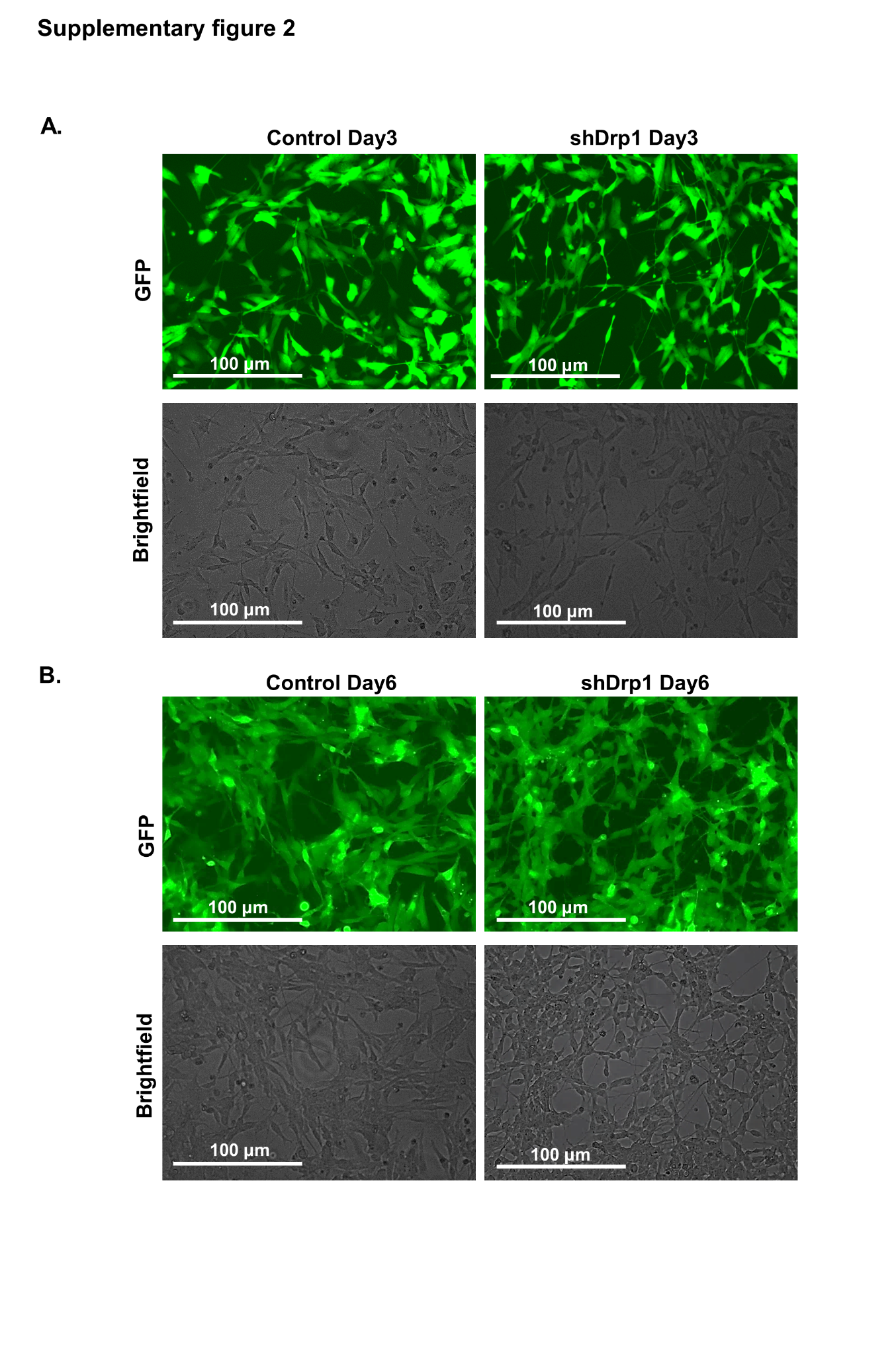
**

**Figure S2. Monitoring neuronal differentiation of control and shDrp1 cells on Days 3 and 6.** Control and shDrp1 cells were treated with 10 μM RA for 3 days and 50 ng/ml BDNF for the next 3 days to achieve neuronal differentiation. Fluorescence (pGIPZ-GFP) and brightfield images of live cells were taken on Days 3 **(A)** and 6 **(B)** using an Invitrogen EVOS Digital Color Fluorescence Inverted Microscope.

**^
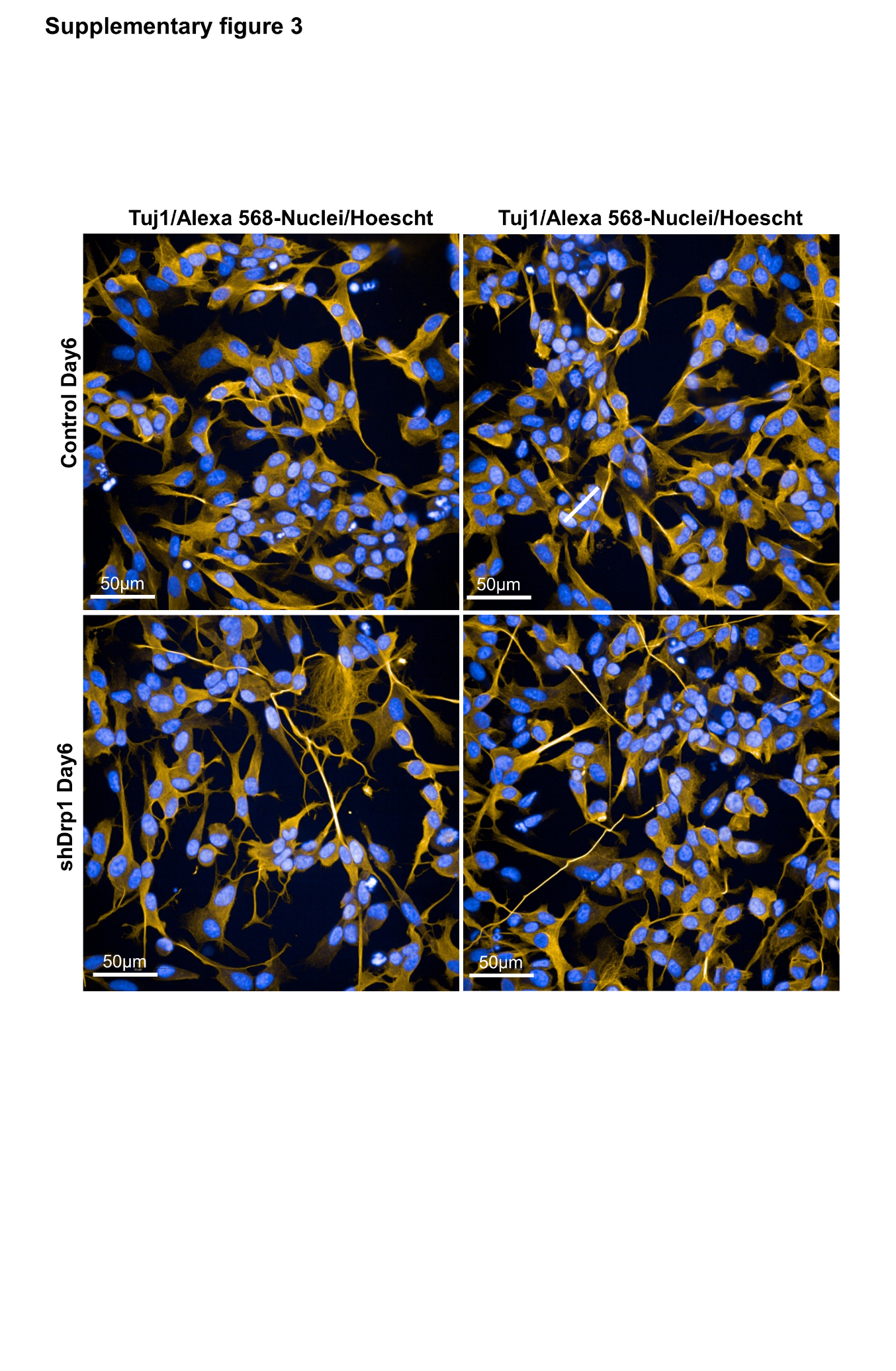
^**

**Figure S3. Confirming neuronal differentiation of control and shDrp1 cells.** Control and shDrp1 cells were treated with 10 μM RA for 3 days and 50 ng/ml BDNF for the next 3 days to achieve neuronal differentiation. Neuron-specific β-tubulin III staining of fixed control and shDrp1 cells at Day 6 of differentiation was performed using an anti-Tuj1 antibody.

**
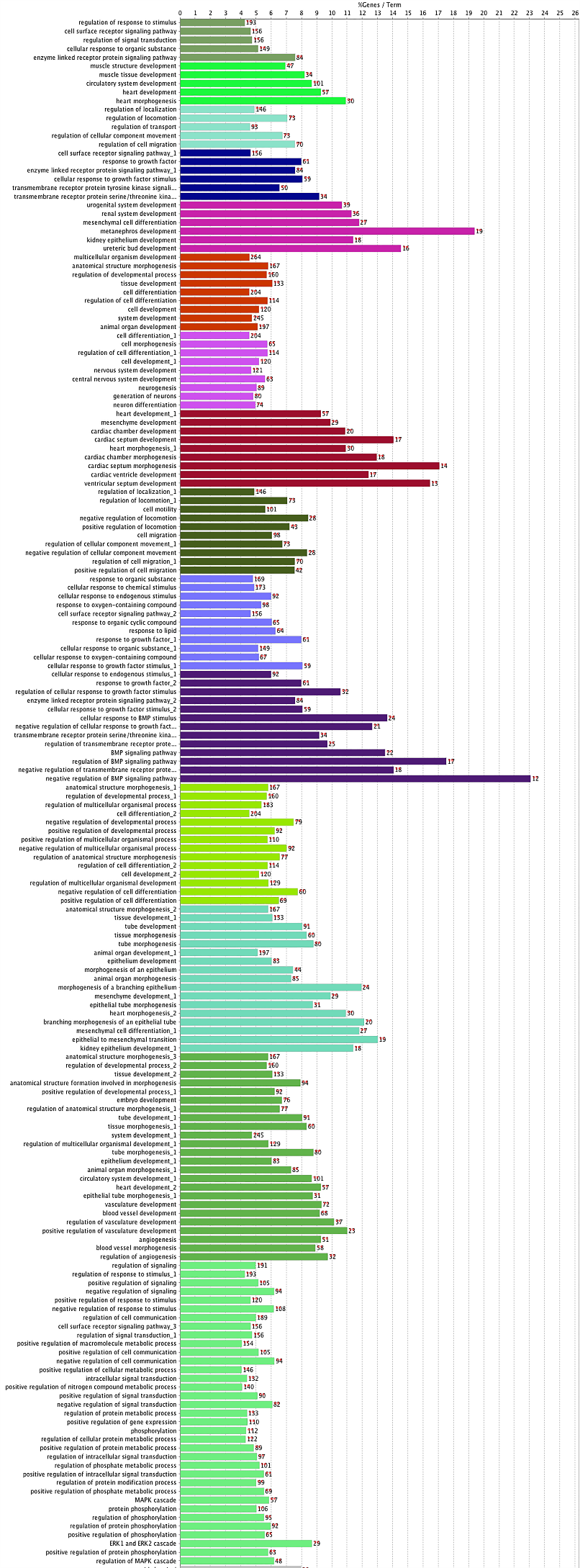
**

**Figure S4. Separate GO analysis of downregulated genes in shDrp1 cells compared to control cells.** Overrepresented GO terms (biological processes) of downregulated genes in shDrp1 cells are shown separately. Bar graphs represent the associated genes' numbers for each significant GO term and %Genes/Term for downregulated genes. CytoScape 3.4.0 with the ClueGo application was used for gene ontology analysis of differentially expressed genes. A hypergeometric test with Bonferroni step-down correction was used to determine overrepresented GO categories.


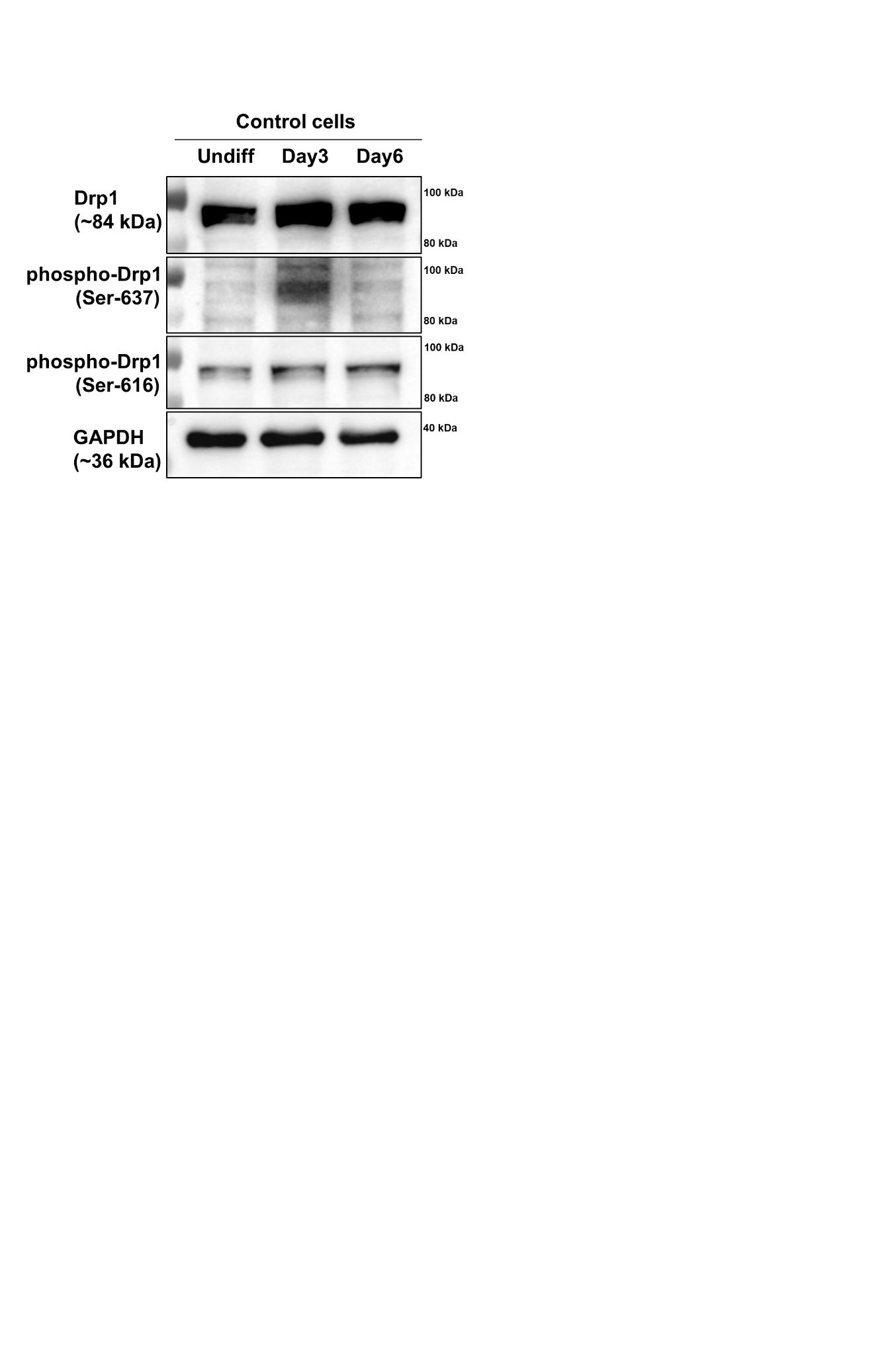


**Figure S5.** Unphosphorylated and phosphorylated Drp1 at Ser-637 and Ser-616 were examined using anti-Drp1 and phospho-specific antibodies in undifferentiated and differentiated (Days 3 and 6) control cells. Cells were lysed with RIPA buffer, and an equal amount of protein was separated. GAPDH was used as an internal loading control. Representative images of western blots are shown.


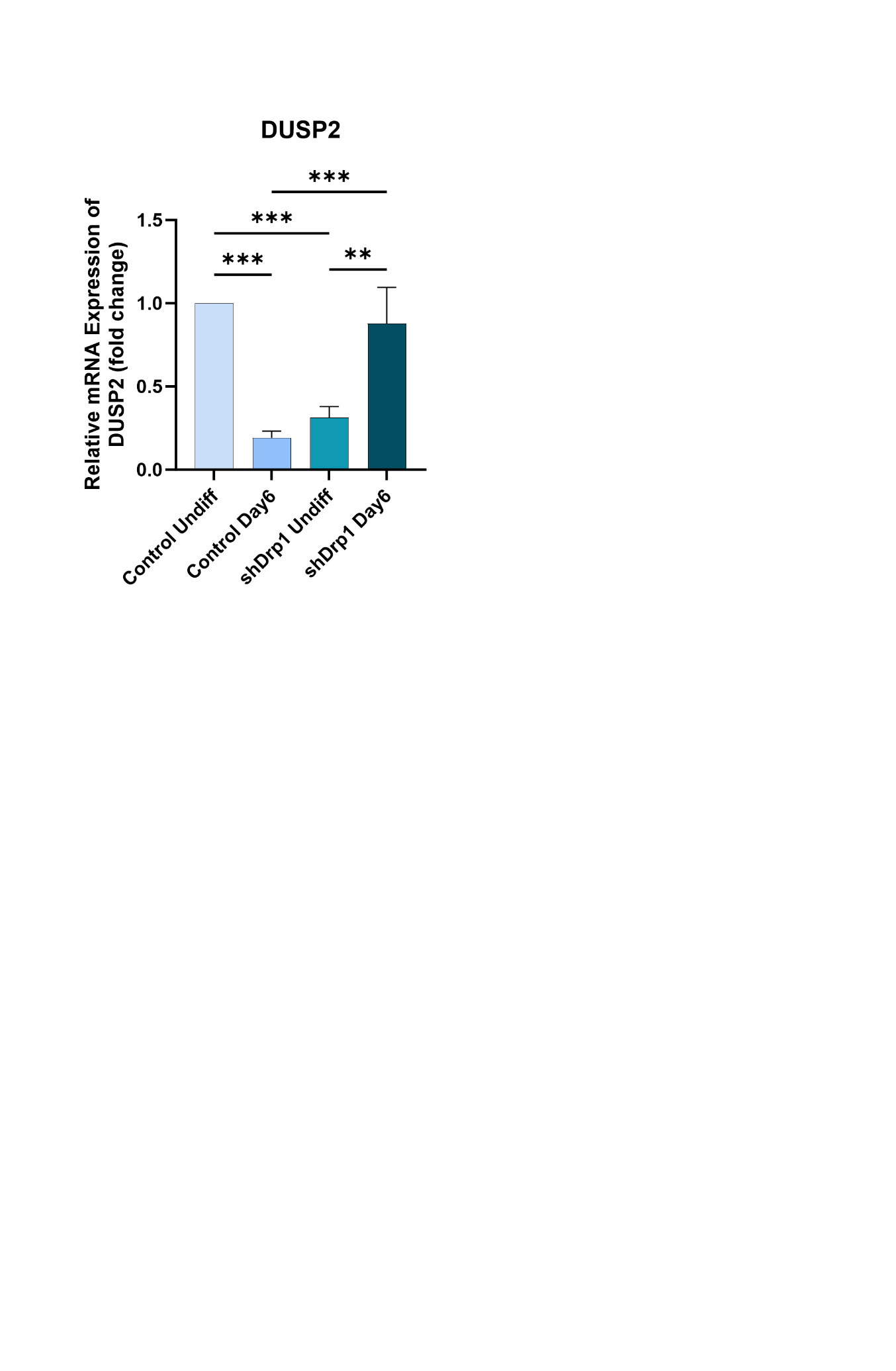


**Figure S6.** DUSP2 mRNA levels were measured using qPCR in undifferentiated and differentiated control and shDrp1 cells. Data are presented as mean values ± SD of n = 5 separate experiments. Statistical analysis was performed by One-way ANOVA (** indicates p < 0.01*** indicates p < 0.001).
